# An Interactive Voice Response Software to Improve the Quality of Life of People Living With HIV in Uganda: Randomized Controlled Trial

**DOI:** 10.2196/22229

**Published:** 2021-02-11

**Authors:** Dathan Mirembe Byonanebye, Maria S Nabaggala, Agnes Bwanika Naggirinya, Mohammed Lamorde, Elizabeth Oseku, Rachel King, Noela Owarwo, Eva Laker, Richard Orama, Barbara Castelnuovo, Agnes Kiragga, Rosalind Parkes-Ratanshi

**Affiliations:** 1 Department of Community Health and Behavioural Sciences Makerere University School of Public Health Makerere University Kampala Uganda; 2 The Academy for Health Innovations Infectious Diseases Institute Makerere University Kampala Uganda; 3 Infectious Diseases Institute Makerere University College of Health Sciences Makerere University Kampala Uganda; 4 Cambridge Institute of Public Health School of Clinical Medicine University of Cambridge Cambridge United Kingdom

**Keywords:** mHealth, HIV, quality of life, interactive voice response, mobile health, digital health

## Abstract

**Background:**

Following the successful scale-up of antiretroviral therapy (ART), the focus is now on ensuring good quality of life (QoL) and sustained viral suppression in people living with HIV. The access to mobile technology in the most burdened countries is increasing rapidly, and therefore, mobile health (mHealth) technologies could be leveraged to improve QoL in people living with HIV. However, data on the impact of mHealth tools on the QoL in people living with HIV are limited to the evaluation of SMS text messaging; these are infeasible in high-illiteracy settings.

**Objective:**

The primary and secondary outcomes were to determine the impact of interactive voice response (IVR) technology on Medical Outcomes Study HIV QoL scores and viral suppression at 12 months, respectively.

**Methods:**

Within the Call for Life study, ART-experienced and ART-naïve people living with HIV commencing ART were randomized (1:1 ratio) to the control (no IVR support) or intervention arm (daily adherence and pre-appointment reminders, health information tips, and option to report symptoms). The software evaluated was Call for Life Uganda, an IVR technology that is based on the Mobile Technology for Community Health open-source software. Eligibility criteria for participation included access to a phone, fluency in local languages, and provision of consent. The differences in differences (DIDs) were computed, adjusting for baseline HIV RNA and CD4.

**Results:**

Overall, 600 participants (413 female, 68.8%) were enrolled and followed-up for 12 months. In the intervention arm of 300 participants, 298 (99.3%) opted for IVR and 2 (0.7%) chose SMS text messaging as the mode of receiving reminders and health tips. At 12 months, there was no overall difference in the QoL between the intervention and control arms (DID=0.0; *P*=.99) or HIV RNA (DID=0.01; *P*=.94). At 12 months, 124 of the 256 (48.4%) active participants had picked up at least 50% of the calls. In the active intervention participants, high users (received >75% of reminders) had overall higher QoL compared to low users (received <25% of reminders) (92.2 versus 87.8, *P*=.02). Similarly, high users also had higher QoL scores in the mental health domain (93.1 versus 86.8, *P*=.008) and better appointment keeping. Similarly, participants with moderate use (51%-75%) had better viral suppression at 12 months (80/94, 85% versus 11/19, 58%, *P*=.006).

**Conclusions:**

Overall, there was high uptake and acceptability of the IVR tool. While we found no overall difference in the QoL and viral suppression between study arms, people living with HIV with higher usage of the tool showed greater improvements in QoL, viral suppression, and appointment keeping. With the declining resources available to HIV programs and the increasing number of people living with HIV accessing ART, IVR technology could be used to support patient care. The tool may be helpful in situations where physical consultations are infeasible, including the current COVID epidemic.

**Trial Registration:**

ClinicalTrials.gov NCT02953080; https://clinicaltrials.gov/ct2/show/NCT02953080

## Introduction

The response to the HIV/AIDS epidemic and the scale-up of antiretroviral therapy (ART) has been successful globally. Over the last decade alone, the number of people living with HIV who are receiving ART increased from 400,000 in 2003 to 24.5 million in 2019 [1]. Although ART improves the quality of life (QoL) in people living with HIV [2,3], studies after ART scale-up continue to report low QoL, even in people living with HIV receiving ART [4]. The World Health Organization (WHO) has highlighted the need for HIV care programs to increase coverage of comprehensive HIV services and ensure good QoL for people living with HIV [5].

The biomedical goal of ART is to stop HIV replication, achieve viral suppression, and ultimately reduce HIV-associated mortality and its transmission. To achieve these goals, people living with HIV need to be highly adherent to ART and remain engaged in care. Ensuring social support and improving knowledge about HIV ART improves the QoL in people living with HIV [3]. Conversely, poor adherence [6] and noncontrolled symptoms [7] are associated with poorer QoL. To this end, HIV care should encourage strict adherence to ART and support symptom surveillance and alleviation to improve the QoL in people living with HIV. However, the provision of continuous adherence support and symptom surveillance is challenging and time-consuming and requires resources. Therefore, there is a need for patient-centered systems that enhance adherence and symptom surveillance but do not burden already-constrained health systems, especially in the low- and middle-income countries (LMICs) with a high burden of HIV.

Following the increasing access to mobile technology in the most-burdened countries [8], mobile health (mHealth) technologies are increasingly leveraged to support people living with HIV. However, there is limited evidence on the impact of mHealth tools on QoL in people living with HIV, especially in LMICs. The majority of mHealth tools evaluated in LMICs have used SMS text messaging interventions with mixed results [9]. The adoption and impact of SMS text messaging interventions are likely to be low in countries with low literacy rates. This study sought to determine the acceptability and impact of an interactive voice response (IVR)–based patient support technology among people living with HIV in Uganda. The IVR technology allows for two-way communication between the software and end user and can be deployed on a simple feature phone. The IVR tool evaluated in this study delivered daily adherence reminders, appointment reminders, and weekly health tips to people living with HIV.

**Figure 1:**
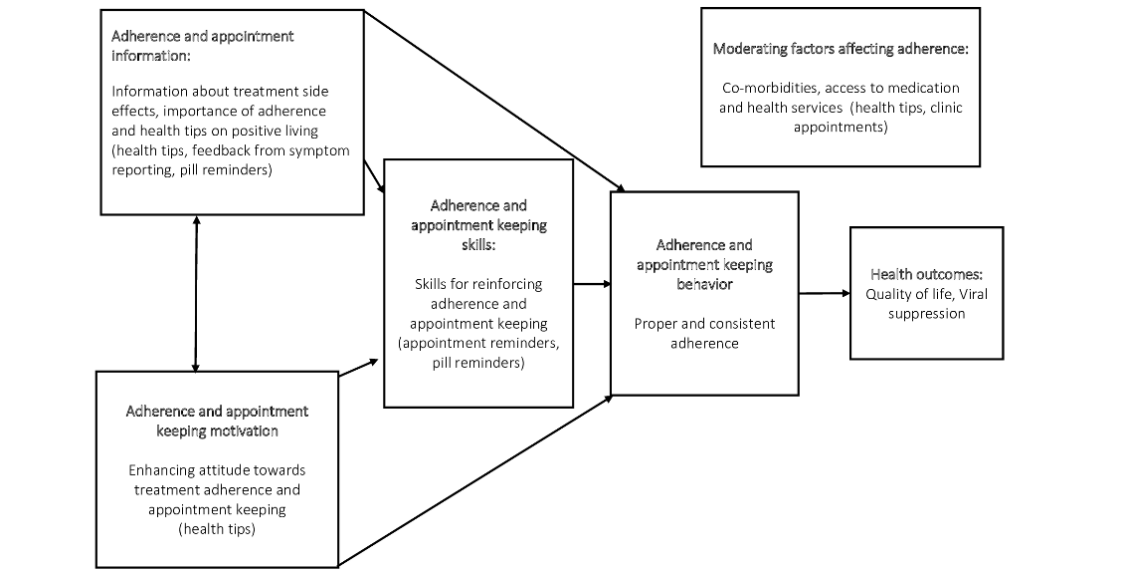
Behavioral change conceptual framework for the study, adapted from the information, motivation, and behavioral skills model [11].

To increase the adoption, replication, and impact of the intervention, we used the criteria and taxonomy suggested by Tabak et al [10] and selected the information, motivation, and behavioral skills (IMB) model of behavioral change [11] as the theory for the intervention (Figure 1). The IMB model was initially developed to understand and change HIV-risky behaviors in developed countries [11]. It has since been widely used in LMICs to assess and improve adherence to ART [12,13]. The model suggests that motivation, skilling, and provision of information on adherence to people living with HIV improve adherence. We hypothesized that IVR-based technology could provide motivational information and reminders to people living with HIV and ultimately improve ART adherence, QoL, and viral suppression. Figure 1 shows the aspects of the IMB model that were adapted to design the intervention used in this study.

## Methods

### Study Design

This was an open-label randomized controlled trial (RCT) to evaluate the impact of a patient support tool, Call for Life Uganda (CFL), on QoL and viral suppression in people living with HIV in Uganda. The primary outcome was the difference in the differences in QoL between the study arms at month 12.

### Study Settings

The study was conducted at two HIV clinics. The Infectious Diseases Institute (IDI) clinic is a specialist urban HIV clinic located within the National Mulago Hospital complex and serves more than 8000 people living with HIV. Kasangati Health Centre IV is a government-owned clinic in peri-urban Kampala and serves approximately 5000 people living with HIV. At both clinics, comprehensive HIV care and treatment services were provided according to the WHO and national guidelines for HIV treatment [14,15]. Nurse counselors physically provide face-to-face patient adherence support during clinic visits with no option for remote support.

The technology evaluated in this study was CFL, a software that is based on open-source Mobile Technology for Community Health (MoTeCH). MoTeCH was initially developed by the Grameen Foundation and the University of Southern Maine with the support of Janssen, the Pharmaceutical Companies of Johnson & Johnson. Before this study, the software was used in India and Ghana [16,17]. The initial software was called Treatment Advice by Mobile Alerts (TAMA) [17]. Following the adaptation of TAMA for use by people living with HIV in Uganda, the local system was named Call for Life.

Before adoption, user acceptability testing (UAT) was undertaken with study staff (medical and information systems) and the TAMA team based at Janssen Global Public Health. Between October 2015 and August 2016, 103 “expert” people living with HIV who had been attending IDI for over 5 years and who had prior participation in research were enrolled on the TAMA software. The purpose of this exercise was to tailor the software to the needs of people living with HIV and care providers in Uganda and to pilot health tip content. Before adding patients to the system, 6 UAT sessions were held to test inbound and outbound calls, the registration process, and the functioning of the audio files. In December 2015, a focus group discussion was held with 43 people living with HIV registered on TAMA to understand the experiences and challenges users faced. The major challenges experienced during study implementation included internet and system failures and the need for security upgrades (Multimedia Appendix 1). As a result, the system was iteratively upgraded from version 1.0.0 to version 9.3.0, before and during use in the study. The changes aimed at optimizing usability and security, not the intervention delivered, as summarized in Multimedia Appendix 2.

The health tips were developed by an experienced team of HIV clinicians and behavior change specialists in collaboration with Straight Talk Foundation, a local nongovernmental organization that specializes in health messaging and behavior change. The health tips were based on international and national best practices and guidelines for HIV messaging [15,18]. In collaboration with Community Health and Information Network Uganda, a patient advocacy nongovernmental organization, focus group discussions were held in January and February 2016 with people living with HIV not enrolled on the tool to assess the acceptability and clarity of the messages. The discussions also assessed the accuracy of translations to local languages. Overall, there were 330 messages that covered various topics including general information on HIV, antiretroviral therapy and adherence, positive living, sexual and reproductive health, pregnancy, and safe breastfeeding and general health. Multimedia Appendix 3 summarizes the message categories and provides examples of each.

### Study Intervention

The software evaluated in this study is CFL and is compatible with messaging in four languages: English, Luganda, Kiswahili. and Runyankore. The system allows automatic interaction with patients using voice and tone input via a keypad (IVR) or by SMS text messaging using simple phones (GSM-2/feature). CFL was integrated with the patient health information management systems used for HIV care in Uganda, so as to obtain appointment dates and ART regimens. Participants in the intervention arm received the usual standard of care plus daily adherence IVR voice reminders (or SMS text messaging), delivered just before the usual pill-taking time. Intervention participants also received pre-appointment reminders and weekly voice calls offering educational health tips. In addition, intervention participants had an option to call a toll-free line and report symptoms or drug side effects. Such patient-triggered calls could generate alerts that would prompt health care workers to call back within 24 hours. Participants chose the preferred languages, time, and frequency of receiving reminders. For security, both outbound and inbound calls played music until the participant entered a personal identification number (PIN) unique to them. Participants chose their preferred “health topics”, from which the system randomly shuffled and randomly played different health tips for each call (Multimedia Appendix 3). Multimedia Appendix 4 shows the call flow diagram for the CFL software. Overall, participants received reminders and had to key in their PIN before listening to the calls. After the reminder was played, the system prompted participants to select additional services they wanted using buttons on the phone keypad. People living with HIV in the control arm received standard of care comprising face-to-face facility appointments but no access to remote adherence, appointment reminders, or symptom reporting.

### Quality Assurance and Software Updates

The health care workers accessed the CFL dashboard on the web [19], which required connection to an internet server (thus needing a stable connection to power and internet). Participants did not require smartphones. They received and made calls using any mobile phones, including feature phones. The software allows for the configuration of call times, inputting of mobile phone contacts, PIN setting, and synchronization of data with the patient health information systems.

At baseline, participants in the intervention arm were trained on how to initiate and receive calls. Participants were asked at each appointment if they were experiencing challenges with the IVR calls. Call completion rates were reviewed weekly. Patients with a blocked PIN were contacted by the study medical team within one week to reset the PIN. Throughout the study period, there were 16 upgrades of the CFL software (Multimedia Appendix 2). However, the content delivered by the software did not change throughout the follow-up period, except for health tips; following feedback from the participants, 30 additional messages (16 on nutrition and 14 on cancer) were added.

### Sample Size and Power

Based on pre- and post-ART assessments of QoL in people living with HIV in Burkina Faso [7] and Uganda [20], we anticipated a 15-point difference in overall QoL following ART in people living with HIV in the control and intervention arms. We also estimated that there would be an additional 5-point improvement in the QoL in those receiving CFL. Therefore, we expected an overall difference of 5 points in ART-experienced people living with HIV in the intervention versus control arms. For a power of 90% and precision of 0.05, we needed a minimum of 273 patients in each arm (overall 546) to detect a 5-point difference in the QoL in the intervention versus the control arm. The estimated sample size was adjusted for the anticipated attrition of 9% to give a final sample size of 600.

### Randomization

Eligible patients were randomized to either the control (standard of care) or intervention arm (1:1 ratio) in this open-label study. Randomization blocks (sizes of 4) were generated by an independent statistician and kept under lock and key at the two sites. The study medical team assigned randomized participants to their final allocated study arms. We interviewed participants in the intervention arm at each visit if they had trouble using the intervention. A detailed trial design can be found in the study protocol (Multimedia Appendix 5).

### Study Procedures and Data Collection

Participants were physically evaluated at baseline and months 6 and 12. At each time point, the study team collected data on sociodemographics (age, sex, marital status) and treatment history (ART status, duration on ART, ART regimen, and HIV RNA). Plasma HIV RNA testing was performed on plasma at months 6 and 12 using the Roche COBAS TaqMan v2.0 HIV-1 assay. Viral suppression was defined as less than 50 copies of HIV RNA per mL. QoL was measured using the HIV version of the Medical Outcomes Study (MOS-HIV) [21] because its local language version has been validated in Uganda [22]. The MOS-HIV measures health-related QoL in 11 areas: health perceptions, bodily pain, physical function, role function, social function, mental function, vitality, health distress, cognitive function, QoL, and health transition. The QoL scores on this scale range from 0 to 100, with higher scores implying better health. Overall scores and individual scores for the physical health summary (PHS) and mental health summary (MHS) domains were calculated. Adherence to appointments was defined as attending appointments within 3 working days of the scheduled visit.

The study was terminated before its conclusion, based on guidance by an independent data safety monitoring board (DSMB), after it was found that there was no difference between study arms at 6 months. The study was closed when all participants had completed at least 12 months (follow-up period range: 12-24 months). The study protocol and DSMB allowed all participants who were willing to continue receiving IVR technology support to do so. Throughout the study, treatment-related data were shared with medical teams. Those with detectable HIV RNAs received appropriate treatment that included intensive adherence counseling.

### Study Subjects

ART-naïve and ART-experienced people living with HIV were consecutively screened, and participants were eligible for enrollment if they belonged to any of the following categories: ART-naïve adults or ART-experienced people living with HIV, including key populations (sex workers and men who have sex with men), young adults (18-24 years), pregnant and breastfeeding mothers, and people living with HIV in discordant relationships. People living with HIV were eligible if they were 18 years or older, were willing to comply with study procedures, and had access to and were able to use a cell phone. Participants also spoke English or one of the available local languages and provided informed consent. We excluded people living with HIV with clinical conditions that could interfere with the use of cell phone (for example, deafness, severe cognitive impairment, critical illness), and those who were not receiving the standard first-line (efavirenz, tenofovir disoproxil fumarate, and lamivudine) or second-line (atazanavir or lopinavir with boosted ritonavir plus lamivudine and tenofovir) ART regimens. Participant screening and enrollment were done in person by study medical teams.

### Statistical Analysis

The primary endpoint was the difference in the change in the QoL (MOS-HIV) at 12 months among ART-experienced people living with HIV in the intervention and control arms at the two study sites. The secondary outcomes were differences in viral suppression (HIV RNA<50 copies/mL) and appointment keeping. All analyses were conducted using Stata software, version 14 (StataCorp). We compared the changes in the QoL and HIV RNA outcomes using Pearson chi-square or paired *t* test and determined the difference in differences (DID) in the endpoints between intervention and control arms. Analysis of covariance was used to test the interaction effects of categorical variables on the QoL, controlling for the effects of other selected continuous variables, including baseline HIV RNA, CD4, and duration of care. Additionally, we compared the outcomes within the intervention arm according to the intensity of use of the system (proportion of users receiving reminders): low users (<25% calls answered), fairly low users (26%-50% of calls answered), moderate users (51%-75% answered) and high users (>75% calls answered). The results of this analysis are reported as per the CONSORT-EHEALTH (Consolidated Standards of Reporting Trials of Electronic and Mobile Health Applications and Online Telehealth) [23] and are also consistent with the mobile health evidence reporting and assessment guidelines (Multimedia Appendix 6) [24].

### Data Security

All study data were double-encrypted by CFL. All clinic data of people living with HIV remained on the local IDI servers as per Uganda data protection guidelines. Communication between the CFL browser and the server was encrypted using 128-bit Secure Sockets Layer. CFL system servers were hosted by Amazon Web Services (AWS) and secured by Amazon virtual private cloud and AWS web firewalls. At the same time, data were protected from virus threats using Bitdefender antivirus technology.

### Ethics

The study was approved by the Makerere University School of Medicine Research Ethics Committee (REC# 2015-083) and Uganda National Council of Science and Technology and was registered with ClinicalTrials.gov (NCT02953080) [25]. All study participants provided informed consent before participation. An independent DSMB supervised the study implementation. An interim analysis was planned at 12 months a priori, and the study was to be terminated if there was no difference in the primary outcomes between study arms at 6 months.

## Results

### Study Population

**Figure 2:**
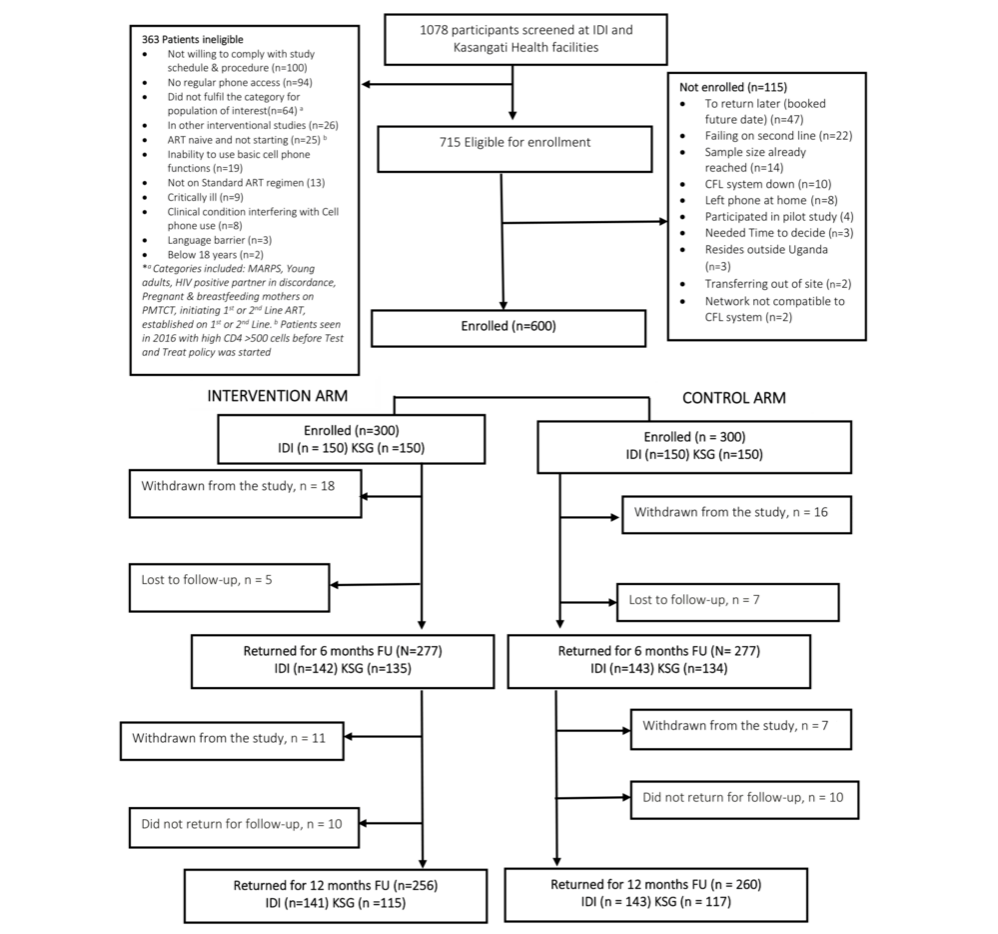
Study enrollment, randomization, and follow-up. ART: antiretroviral therapy; CFL: Call for Life; FU: follow-up; IDI: Infectious Diseases Institute; KSG: Kasangati Health Centre IV; MARPS: most-at-risk populations; PMTCT: prevention of mother-to-child transmission.

From August 2016 to August 2017, 1079 participants were screened concurrently at the two study clinics, 715 participants were eligible, and 600 participants were enrolled (Figure 2). The most common reasons for nonenrollment included postponing enrollment to a date beyond the enrollment period—such participants were not enrolled as they returned after the sample size had been accrued (47)—and failing on second-line ART (22). The other reasons for exclusion are shown in Figure 2. 

Of the 600 enrolled participants (300 in each arm), 554 completed 6 months (277, 92.3% in each arm), and 516 completed 12 months (256, 85.3% and 260, 86.7% in the intervention and control arms, respectively). Of the 600 enrolled participants, 413 (68.8%) were female, 468 (78%) were receiving first-line ART, and 388 (64.7%) had undetectable HIV RNA (Table 1). The median age (IQR) was 32 (25-40) years, one-third (193/600, 32.2%) were in serodiscordant relationships, and 155 (25.8%) were pregnant or breastfeeding.

**Table 1:** Characteristics of study participants (N=600).

Characteristic			Total (N=600)		Intervention (n=300)		Standard (n=300)		*P* value^a^
**Gender, n (%)**									
	Female	413 (68.8)		210 (70.0)		203 (67.7)		.54	
	Male	187 (31.2)		90 (30.0)		97 (32.3)		—^b^	
**Age (years), n (%)**									
	16-24	161 (26.8)		82 (27.3)		79 (26.3)		.78	
	25-35	219 (36.5)		104 (34.7)		115 (38.3)		—	
	36-50	181 (30.2)		95 (31.7)		86 (28.7)		—	
	≥50	39 (6.5)		19 (6.3)		20 (6.7)		—	
**Marital status, n (%)**									
	Married	449 (74.8)		223 (74.3)		226(75.3)		.78	
	Not married	151 (25.2)		77 (25.7)		74 (24.7)		—	
**Education level, n (%)**									
	None	24 (4.0)		8 (2.7)		16 (5.3)		.32	
	Primary	231 (38.5)		118 (39.3)		113 (37.7)		—	
	Secondary	265 (44.2)		137 (45.7)		128 (42.7)		—	
	Tertiary	80 (13.3)		37 (12.3)		43 (14.3)		—	
**Alcohol use, n (%)**									
	Yes	292 (48.7)		147 (49.3)		144 (48.0)		.74	
	No	308 (51.3)		152(50.7)		156(52.0)		—	
**ART^c^ regimen, n (%)**									
	First-line	468 (78.0)		230 (76.7)		238 (79.3)		.43	
	Second-line	132 (22.0)		70 (23.3)		62 (20.7)		—	
Duration on ART (years), median (IQR)			2.0 (0.3-3.8)		1.8 (0.3-3.8)		2.1 (0.3-3.7)		.23
**Baseline HIV RNA, n (%)**									
	HIV RNA≥50 copies/mL	212 (35.3)		113 (37.3)		99 (33.0)		.28	
	HIV RNA<50 copies/mL	388 (64.7)		187 (62.7)		201 (67.0)		—	

^a^There were no differences between the two study arms, and *P* was attained using the Pearson chi-square test.

^b^Not available.

^c^ART: antiretroviral therapy.

### Change in Quality of Life

The mean overall MOS-HIV QoL scores at baseline in the intervention and control arms were 85.5 and 86.0, respectively, and this increased to 90.3 and 90.7, respectively, at 12 months (DID=0.0, SE 1.03; *F*=0.52*, P*=.47) (Table 2). The change in PHS domain score between baseline and months was also comparable between the two arms (DID=−0.1, SE 1.28; *F*=0.600, *P*=.44). Similarly, there was no overall difference in the change in MHS QoL scores between the two study arms (DID=0.2; *F*=0.860, *P*=.35). Subanalyses did not show any significant difference between arms in any category except when the analysis was stratified by the intensity of the use of the software.

**Table 2:** Quality of life scores at baseline and 12 months.

QoL^a^ domain and subgroup		Baseline					Follow-up (month 12)					DID^b^ analysis			ANCOVA^c^ analysis	
		I^d^, mean	C^e^, mean	Diff^f^ (I-C)	*P* value	I, mean		C, mean	Diff (I-C)	*P* value	DID		*P* value	*F*		*P* value
**MOS-HIV^g^**																
	Overall	85.5	86.0	−0.5	.53	90.3		90.7	−0.5	.54	0.0		.99	0.52		.47
	IDI^h^	89.3	90.7	−1.5	.04	90.9		92.1	−1.2	.10	0.3		.80	3.14		.08
	KSG^i^	80.9	80.1	0.8	.51	89.6		89.1	0.5	.67	−0.3		.87	0.16		.69
**Mental health summary score**																
	Overall	86.6	87.4	−0.8	.30	91.5		92.1	−0.6	.46	0.2		.83	0.86		.35
	IDI	90.8	92.2	−1.4	.07	92.3		93.9	−1.6	.04	−0.2		.86	6.26		.01
	KSG	81.4	81.4	−0.1	.94	90.5		89.8	0.7	.58	0.8		.66	0.54		.46
**Physical health summary score**																
	Overall	86.7	87.3	−0.6	.52	92.3		93.0	−0.7	.45	−0.1		.93	0.60		.44
	IDI	90.3	92.2	−1.9	.06	92.7		94.0	−1.3	.19	0.6		.67	1.24		.27
	KSG	82.4	81.4	1.0	.48	91.8		91.7	0.1	.96	−1.0		.64	0.01		.91

^a^QoL: quality of life.

^b^DID: difference in differences; the difference between the differences in QoL scores between the intervention and control arms at baseline and 12 months.

^c^ANCOVA: analysis of covariance.

^d^I: intervention arm.

^e^C: control arm.

^f^Diff: difference between scores at baseline or 12 months.

^g^MOS-HIV: Medical Outcomes Study, HIV version.

^h^IDI: Infectious Diseases Institute.

^i^KSG: Kasangati Health Centre IV.

### Change in HIV RNA at 12 Months

At baseline, viral suppression rates in the intervention and control arms were 62.7% (188/300) and 67% (201/300), respectively. At month 12, viral suppression improved to 80.9% (195/241) and 82.6% (213/258) among participants who had data on HIV RNA in the intervention and control arms, respectively. There was no difference between the intervention and control arms regarding the change in the log HIV RNA between baseline and 6 months (DID=0.05, SE 0.137, *P*=.66) and at 12 months (DID=0.01, SE 0.134, *P*=.94). Similarly, there was no difference in the change in log HIV RNA between the two arms (Table 3).

**Table 3:** Mean percentage scores of HIV RNA log copies/mL by arm and study group.

Time point	HIV RNA (copies/mL)														
	Baseline						Follow-up						DID^a^		
	I, mean^b^	C, mean^c^	Diff^d^ (I-C)	SE	*P* value	I, mean		C, mean	Diff I-C)	SE	*P* value	DID		SE	*P* value
6 months	2.04	2.09	−0.05	0.094	.63	1.66		1.66	0.00	0.099	.88	0.05		0.137	.66
12 months	2.02	2.02	0.00	0.094	.95	1.67		1.66	0.01	0.095	.87	0.01		0.134	.94

^a^DID: difference in differences; the difference between the differences in log HIV RNA copies between the intervention and control arms at baseline and 6 and 12 months.

^b^I: intervention arm.

^c^C: control arm.

^d^Diff: difference in the log HIV RNA copies between the I and C arms.

### Adherence to Appointments

Overall, there was significantly higher adherence to appointments (*P*=.04) in the participants in the intervention arm at 6 months (200/277, 72.2%), compared to those in the control arm (178/277, 64.3%). However, at 12 months, there was no difference in appointment keeping in the intervention versus the control arm (intervention arm: 178/256, 69.5%; control arm: 178/260, 68.5%; *P*=.79).

### Fidelity to Intervention Delivery

**Figure 3:**
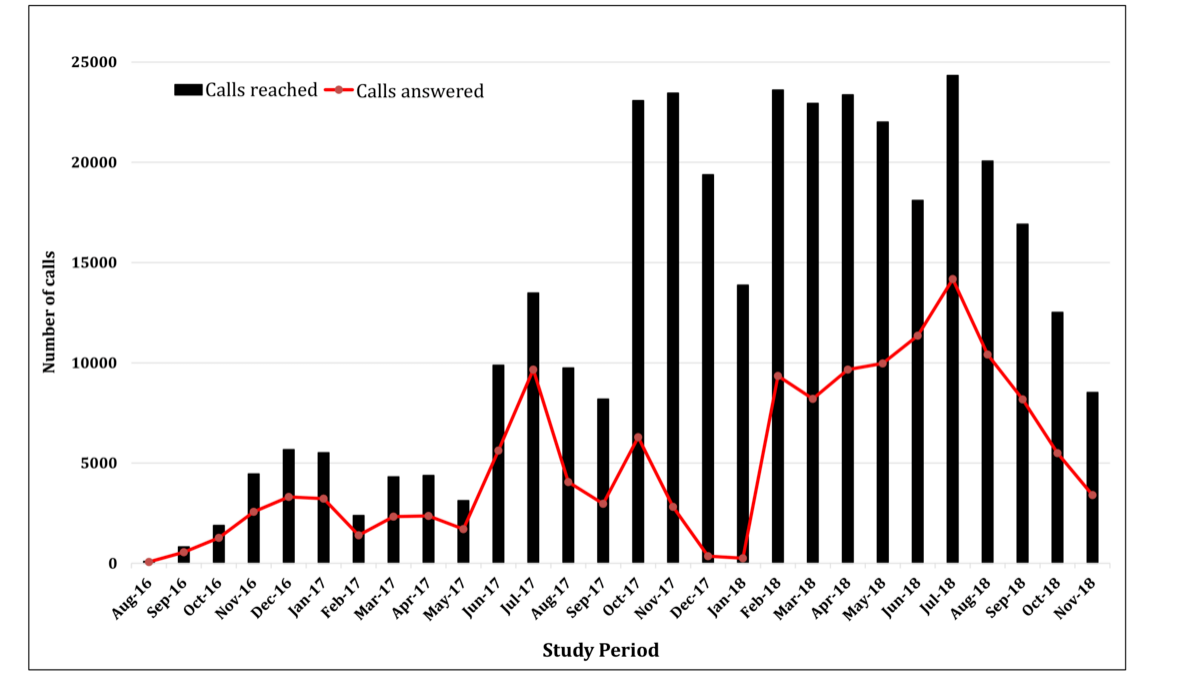
Number of calls made and successfully completed by the study participants in the intervention arms.

At baseline, 298 of the 300 participants (99.7%) in the intervention arm chose IVR, and only 2 selected SMS text messaging. Throughout the study, 346,286 outbound calls were made, of which 182,943 (52.8%) calls were answered and 141,043 (40.7%) were uninterrupted until the end (Figure 3). At 12 months, 25 of the 256 active participants (9.8%) were low users, 107 (41.8%) were moderate users, and 124 (48.4%) were high users.

The study was halted between January 20 and February 2, 2018, due to a software failure. There were no confidentiality breaches during this software failure, and a protocol deviation was reported to the institutional review board. Upon fixing the technical failure, participants were interviewed on whether they wanted to re-enroll. Only 1 participant out of 299 patients on the intervention arm declined (due to reasons other than software) to rejoin the study after this time. During the system failure period, all participants received the standard of care.

### Outcomes by the Level of Use of the Tool

Among participants in the intervention arm who were active at 12 months, there was a general trend toward better study outcomes in participants with higher use of software compared to low users (received <25% of all reminder calls). The baseline scores were generally similar in the different strata of tool usage (Table 4). At 12 months, overall QoL, as well as the QoL scores in the MHS domain, was higher for participants with high use of the tool than for those with low use (overall QoL score: 92.2 versus 87.8, *P*=.02; MHS scores: 93.1 versus 90.7, *P*=.008). However, there was no statistical difference in the PHS domain scores between higher and low users (94.6 versus 90.7, *P*=.07) (Table 4).

**Table 4:** Change in quality of life by utilization of the intervention.

QoL^a^ domain, by calls answered (%)			Baseline							6-month follow-up							12-month follow-up				
			Participants, n		QoL scores, mean (SD)		*P* value^b^		Participants, n			QoL scores, mean (SD)		*P* value^b^		Participants, n			QoL scores, mean (SD)		*P* value^b^
**MOS-HIV^c^**																					
	0-25	37		85.2 (7.6)		Ref^d^		58			88.5 (7.2)		Ref		25			87.8 (8.2)		Ref	
	26-50	123		86.1 (8.7)		.57		88			88.5 (7.1)		.96		107			90.8 (5.1)		.04	
	51-75	108		83.8 (10.9)		.52		92			88.2 (8.8)		.79		99			89.9 (7.0)		.24	
	76-100	30		90 (7.1)		.02		39			89.4 (5.7)		.54		25			92.2 (3.2)		.02	
**Mental health score**																					
	0-25	37		86.1 (8.4)		Ref		58			89.5 (7.1)		Ref		25			86.8 (10.3)		Ref	
	26-50	123		86.7 (9.9)		.76		88			89.9 (6.3)		.73		107			91.9 (5.1)		.001	
	51-75	108		85.3 (11.6)		.72		92			90.0 (7.7)		.68		99			91.6 (6.1)		.005	
	76-100	30		90.5 (8.8)		.06		39			91.5 (4.9)		.14		25			93.1 (4.1)		.008	
**Physical health score**																					
	0-25	37		86.5 (9.6)		Ref		58			90.8 (10.1)		Ref		25			90.7 (8.6)		Ref	
	26-50	123		88.1 (9.7)		.39		88			90.7 (10.4)		.96		107			92.9 (7.1)		.19	
	51-75	108		84.1 (13.1)		.34		92			89.2 (12.6)		.41		99			91.4 11.0)		.79	
	76-100	30		92.1 (7.8)		.02		39			90.3 (8.7)		.77		25			94.6 (5.4)		.07	

^a^QoL: quality of life.

^b^The *P* values are attained using one-way analysis of variance.

^c^MOS-HIV: Medical Outcomes Study, HIV version.

^d^Ref: reference group for comparisons.

Compared to low users, viral suppression rates at 12 months were higher in those with higher usage. This was significant for those with moderate usage of the tool (80/94, 85% versus 11/19, 58%; *P*=.006) and in those with fairly low usage (26%-50% usage) (84/103, 81.5% versus 11/19, 57.9%; *P*=.02). However, the difference did not reach significance in those with highest usage in viral suppression (20/25, 80% versus 11/19, 58%; *P*=.11) (Table 5).

Participants with better usage of the tool also generally had higher rates of appointment keeping compared to low users. Compared to the rates in low users (12/25, 48%), appointment keeping was higher in participants with fairly low usage (74/107, 69.2%, *P*=.046), moderate usage (72/99, 73%, *P*=.02), and high usage (20/25, 80%, *P*=.02) (Table 5).

**Table 5:** Viral suppression and appointment keeping at 12 months by the level of use of the tool.

Outcome, by calls answered (%)			Month 6				Month 12		
			Participants, n/N (%)^a^		*P* value^b^		Participants, n/N (%)^c^		*P* value^b^
**Viral suppression^d^**									
	0-25	49/57 (86.0)		Ref^e^		11/19 (57.9)		Ref	
	26-50	64/86 (74.4)		.16		84/103 (81.6)		.02	
	51-75	74/90 (82.2)		.75		80/94 (85.1)		.006	
	76-100	32/39 (82.1)		.78		20/25 (80.0)		.11	
**Appointment adherence**									
	0-25	39/58 (67.2)		Ref		12/25 (48.0)		Ref	
	26-50	63/88 (71.6)		.57		74/107 (69.2)		.046	
	51-75	66/92 (71.7)		.56		72/99 (72.7)		.02	
	76-100	33/39 (84.6)		.05		20/25 (80.0)		.02	

^a^5 missing values at 6 months.

^b^The *P* values are attained using the Pearson chi-square test. Missing values not included in the analysis.

^c^16 missing values at 12 months.

^d^HIV RNA <50 copies/mL.

^e^Ref: reference group for comparisons.

### Serious Adverse Events

There were 8 adverse events in each study arm. The adverse events included 7 deaths and 9 hospitalizations. Of the adverse events, 11 were HIV-related, and none were attributed to the use of the tool.

## Discussion

### Principal Findings

Despite the increasing data from pilot studies in LMICs that support mHealth interventions, very few interventions have involved IVR. Similarly, few studies have assessed the scalability and sustainability of mHealth interventions. In this RCT, we determined the impact of an IVR-based patient-centered tool on the QoL and treatment outcomes of people living with HIV. To our knowledge, this is the largest mHealth intervention trial to evaluate the impact of IVR technology and the only one to offer a choice of IVR and SMS text messaging in Africa. Mobile health technologies that utilize voice calls are ideal for patient support in most LMICs due to high illiteracy rates [26]. Our study did not find any statistical difference in the change in QoL at 12 months in participants enrolled in the intervention and control arms. Similarly, there was no difference in viral suppression rates in the two arms. However, we found an association between improved QoL (overall and MHS) as well as viral suppression and adherence to clinic appointments in participants who had moderate or high use of the tool.

Before this study, studies involving SMS-based interventions had reported no improvement in the QoL in people living with HIV [27]. However, studies involving live phone calls to people living with HIV reported significant improvement in the QoL [28]. The CFL software provided automated IVR calls with improvement in QoL in the users. Therefore, it seems that “live calls” and IVR, which is a prerecorded voice, are more effective than SMS text messaging in improving QoL. Other IVR-based tools have previously reported higher ART adherence rates among patients receiving IVR adherence reminders in people living with HIV in comparable resource-limited settings [17,29]. Qualitative interviews suggested that people living with HIV became attached to the “voice” and felt as if they were better cared for.

### Study Limitations

This study has a few limitations. While attempting to increase the generalizability of our study findings, we enrolled a heterogeneous population. Participants included highly ART-experienced people living with HIV as well as those newly diagnosed and initiated on ART. Owing to the successful ART scale-up, people living with HIV commencing ART are increasingly healthier at diagnosis [30,31]. Similarly, ART-experienced people living with HIV may have higher QoL scores. The overall baseline QoL scores in this study were high (86 in each arm), so the study may not have been well powered to detect small differences in QoL or HIV RNA. While viral suppression rates at 12 months had improved in both arms, the heterogeneity of the population limited our ability to show a statistical change in viral suppression. The baseline viral suppression rate was 67%, lower than the rates reported in most HIV programs [32], due to the inclusion of those with detectable viral loads such as ART-naïve people and those failing on first-line therapy. This study was conducted at two different facilities, one urban and the other peri-urban. The QoL of patients attending the peri-urban facility was lower at baseline compared to that of patients attending the urban HIV clinic. This difference narrowed at 12 months. The enrollment of patients at the two facilities introduces heterogeneity in the study population but increases the generalizability of the study results. In addition, access to a simple mobile phone was required to access the tool. There is a gender gap in the accessibility of mobile phones in sub-Saharan Africa, where 15% fewer females own a mobile phone, compared to males [33]. We found that 8.7% (94/1078) of those screened had no access to phones; phone ownership was higher than the 70% national coverage [34]. The high mobile phone ownership is attributable to the predominantly urban and educated study population. In key groups, providing a cheap mobile phone (under US $10) may mitigate this. We will explore the feasibility of this approach in our ongoing study with CFL in youths and adolescents with HIV. Additionally, we did not assess the attentiveness of the participants during the whole call, which is a further limitation. Therefore, some participants may have not listened to the entire health tips and reminders. Focus group discussions and in-depth interviews were undertaken to understand the personal, technological, and environmental determinants of tool use, including among young adults [35]. Participants generally responded that the calls reminded them to take their ART, but some did not pick up the call as they felt that just hearing the ringtone was enough of a reminder. The health tip topic for each participant was changed at each appointment, and with 329 tips available, there was considerable new health tip content. Many participants requested to receive health tips on weekends so that they had time to listen to the health tips. The details of the patient preferences for health tips have been described elsewhere [36]. The greatest challenges expressed were PIN code issues and timing of the calls when busy at work, rather than lack of interest in the content.

### Acceptability and Use of the Tool

Technology problems could have contributed to the results, as the intervention was halted for 3 weeks. Still, after the tool failure, surprisingly 99.7% (299/300) of participants agreed to come back to the study, so technology failure did not seem to affect long-term uptake. The study population was urban and highly educated, and participants may have had alternative adherence reminders, including self-set phone alarms and email alerts. However, we did not inquire about the existence of alternative adherence reminders. Nevertheless, Musiimenta et al documented the lack of adherence reminders as a crucial barrier to adherence to long-term treatment in patients with tuberculosis in Uganda [37].

The proportion of patients who successfully received calls was relatively stable throughout the study period. This is reassuring given that some studies have reported a decline in the utilization of mobile health interventions over time. Since the conclusion of this RCT, the scale-up of CFL has been successful. More than 3000 people living with HIV at three health facilities in Kampala are receiving IVR-based patient support; over 1,300,000 successful calls have been placed using the tool over four years [38]. This study demonstrated a higher preference for IVR over SMS text messaging. Qualitative work suggested that this was due to the belief that confidentiality was greater with an anonymous call compared to a message flashed on a screen, but also due to comfort in hearing a real human voice [35]. This raises the opportunity of using IVR technology to mitigate high illiteracy rates in resource-limited settings. In India, a study reported a higher preference for IVR compared to SMS text messaging reminders [17,39].

The study showed high acceptability of the tool, and those who engaged with the tool had better outcomes. The call success rates (141,043/346,286, 40.7% of all calls) for daily uptake (calls repeated up to 3 times on each day until picked up) are higher than what has been reported in similar settings (range: 22%-31%) [40,41]. For those with high use of the tool, the QoL scores were higher than for those with low use of the tool. This could either mean that this population is keen to engage with care and any support that is given or that the subset who used the tool did have an improvement in outcomes. In LMICs, with the need to provide differentiated HIV services, CFL and IVR solutions could support differentiated care models. The WHO recommends low-intensity engagement for stable people living with HIV to allow resources for those who have or are at risk of viral failure. Appropriate use of IVR could provide reassurance to health facilities that longer periods between appointments and fewer face-to-face sessions are safe for stable patients. In the prevailing circumstances when most LMICs are under lockdown and most people living with HIV do not have physical support, IVR could provide alternative support. The impact and cost-effectiveness of replacing face-to-face consultations with IVR in the COVID era will be assessed in our ongoing research project.

### Conclusion

While this study did not find an overall difference in QoL or viral suppression in people living with HIV, the impact of this software in ART-naïve patients with advanced HIV ought to be determined. The software did not find a significant difference in appointment keeping, but most of the patients were highly experienced. Therefore, the intervention should be evaluated in people living with HIV who are newly engaging in care. This study provides useful information on the feasibility and impact of IVR intervention on QoL of people living with HIV in sub-Saharan Africa. While there was no overall difference in QoL and viral suppression in the two study arms, high-intensity users of the tool showed improvement in QoL and viral suppression. With the declining resources available to HIV programs in Africa and the increasing number of people living with HIV accessing ART, other IVR could be useful to enhance patient support for those that are keen and willing to use the system to support them at home. It could also be used to support people living with HIV who cannot or do not want to attend facilities face-to-face during the COVID epidemic.

## Appendix

Multimedia Appendix 1 Issues identified with TAMA/Call for Life system during the pilot phase.

Multimedia Appendix 2 Version changes and system upgrades of Call for Life Uganda.

Multimedia Appendix 3 Health educational messages (tips) used in the study.

Multimedia Appendix 4 Call flow diagram for Call for Life™ Project.

Multimedia Appendix 5 Call for Life study protocol.

Multimedia Appendix 6 CONSORT-eHEALTH checklist (V 1.6.1) and compliance with the mHealth evidence reporting and assessment (mERA) guidelines.

## Abbreviations

ART: antiretroviral therapy
CFL: Call for Life
DID: difference in differences
DSMB: data safety monitoring board
HIV: human immunodeficiency virus
IDI: Infectious Diseases Institute
IMB: information, motivation, and behavioral skills
IVR: interactive voice response
LMICs: low- and middle-income countries
mHealth: mobile health
MHS: mental health summary
MoTeCH: Mobile Technology for Community Health
MOS-HIV: Medical Outcomes Study, HIV version
PHS: physical health summary
PIN: personal identification number
QoL: quality of life
RCT: randomized controlled trial
TAMA: Treatment Advice by Mobile Alerts
UAT: user acceptability testing
WHO: World Health Organization

## References

[1] (2020). 2019 Global HIV Statistics.

[2] Lifson AR, Grund B, Gardner EM, Kaplan R, Denning E, Engen N, Carey CL, Chen F, Dao S, Florence E, Sanz J, Emery S, INSIGHT START Study Group (2017). Improved quality of life with immediate versus deferred initiation of antiretroviral therapy in early asymptomatic HIV infection. AIDS.

[3] Mannheimer SB, Matts J, Telzak E, Chesney M, Child C, Wu AW, Friedland G, Terry Beirn Community Programs for Clinical Research on AIDS (2005). Quality of life in HIV-infected individuals receiving antiretroviral therapy is related to adherence. AIDS Care.

[4] Miners A, Phillips A, Kreif N, Rodger A, Speakman A, Fisher M, Anderson J, Collins S, Hart G, Sherr L, Lampe FC, ASTRA (Antiretrovirals‚ Sexual Transmission and Attitudes) Study (2014). Health-related quality-of-life of people with HIV in the era of combination antiretroviral treatment: a cross-sectional comparison with the general population. Lancet HIV.

[5] World Health Organization Building an Investment Case. Global Health Sector Strategy on HIV, 2016–2021: Towards Ending AIDS.

[6] Corless Inge B, Voss Joachim, Guarino A J, Wantland Dean, Holzemer William, Jane Hamilton Mary, Sefcik Elizabeth, Willard Suzanne, Kirksey Kenn, Portillo Carmen, Rivero Mendez Marta, Rosa Maria E, Nicholas Patrice K, Human Sarie, Maryland Mary, Moezzi Shahnaz, Robinson Linda, Cuca Yvette (2013). The impact of stressful life events, symptom status, and adherence concerns on quality of life in people living with HIV. J Assoc Nurses AIDS Care.

[7] Jaquet Antoine, Garanet Franck, Balestre Eric, Ekouevi Didier K, Azani Jean Claude, Bognounou René, Dah Elias, Kondombo Jean Charlemagne, Dabis François, Drabo Joseph (2013). Antiretroviral treatment and quality of life in Africans living with HIV: 12-month follow-up in Burkina Faso. J Int AIDS Soc.

[8] (2019). Sub-Saharan Africa will have over 600 million unique subscribers by 2025. The Mobile Economy Sub-Saharan Africa 2019.

[9] Demena BA, Artavia-Mora L, Ouedraogo D, Thiombiano BA, Wagner N (2020). A Systematic Review of Mobile Phone Interventions (SMS/IVR/Calls) to Improve Adherence and Retention to Antiretroviral Treatment in Low-and Middle-Income Countries. AIDS Patient Care STDS.

[10] Tabak RG, Khoong EC, Chambers DA, Brownson RC (2012). Bridging research and practice: models for dissemination and implementation research. Am J Prev Med.

[11] Fisher W, Fisher J, Harman J, DiClemente Ralph J, Crosby Richard A, Kegler Michelle C (2002). The information-motivation-behavioral skills model. Emerging theories in health promotion practice and research: Strategies for improving public health.

[12] Simon MD, Altice FL, Moll AP, Shange M, Friedland GH (2010). Preparing for highly active antiretroviral therapy rollout in rural South Africa: an assessment using the information, motivation, and behavioral skills model. AIDS Care.

[13] Rongkavilit C, Naar-King S, Kaljee L, Panthong A, Koken J, Bunupuradah T, Parsons J (2010). Applying the information-motivation-behavioral skills model in medication adherence among Thai youth living with HIV: a qualitative study. AIDS Patient Care STDS.

[14] World Health Organization (2018). Updated recommendations on first-line and second-line antiretroviral regimens and post-exposure prophylaxis and recommendations on early infant diagnosis of HIV: interim guidelines. Supplement to the 2016 consolidated guidelines on the use of antiretroviral drugs for treating and preventing HIV infection.

[15] Uganda Ministry of Health (2018). Consolidated Guidelines for the Prevention and Treatment of HIV and AIDS in Uganda.

[16] Macleod Bruce, Phillips J, Stone A, Walji A, Awoonor-Williams J (2012). The Architecture of a Software System for Supporting Community-based Primary Health Care with Mobile Technology: The Mobile Technology for Community Health (MoTeCH) Initiative in Ghana. Online J Public Health Inform.

[17] Swendeman D, Jana S, Ray P, Mindry D, Das M, Bhakta B (2015). Development and Pilot Testing of Daily Interactive Voice Response (IVR) Calls to Support Antiretroviral Adherence in India: A Mixed-Methods Pilot Study. AIDS Behav.

[18] World Health Organization (2006). Flipchart for Patient Education: HIV prevention, treatment and care.

[19] Connect for Life Software.

[20] Stangl AL, Wamai N, Mermin J, Awor AC, Bunnell RE (2007). Trends and predictors of quality of life among HIV-infected adults taking highly active antiretroviral therapy in rural Uganda. AIDS Care.

[21] Wu AW, Revicki DA, Jacobson D, Malitz FE (1997). Evidence for reliability, validity and usefulness of the Medical Outcomes Study HIV Health Survey (MOS-HIV). Qual Life Res.

[22] Stangl Anne L, Bunnell Rebecca, Wamai Nafuna, Masaba Humphrey, Mermin Jonathan (2012). Measuring quality of life in rural Uganda: reliability and validity of summary scores from the medical outcomes study HIV health survey (MOS-HIV). Qual Life Res.

[23] Eysenbach G, CONSORT-EHEALTH Group (2011). CONSORT-EHEALTH: improving and standardizing evaluation reports of Web-based and mobile health interventions. J Med Internet Res.

[24] Agarwal S, LeFevre AE, Lee J, L'Engle K, Mehl G, Sinha C, Labrique A (2016). Guidelines for reporting of health interventions using mobile phones: mobile health (mHealth) evidence reporting and assessment (mERA) checklist. BMJ.

[25] Improving Outcomes in HIV Patients Using Mobile Phone Based Interactive Software Support.

[26] Krah EF, de Kruijf JG (2016). Exploring the ambivalent evidence base of mobile health (mHealth): A systematic literature review on the use of mobile phones for the improvement of community health in Africa. Digit Health.

[27] Mbuagbaw L, Thabane L, Ongolo-Zogo P, Lester RT, Mills E, Volmink J, Yondo D, Essi MJ, Bonono-Momnougui R, Mba R, Ndongo JS, Nkoa FC, Ondoa HA (2011). The Cameroon mobile phone SMS (CAMPS) trial: a protocol for a randomized controlled trial of mobile phone text messaging versus usual care for improving adherence to highly active anti-retroviral therapy. Trials.

[28] Huang D, Sangthong R, McNeil E, Chongsuvivatwong V, Zheng W, Yang X (2013). Effects of a Phone Call Intervention to Promote Adherence to Antiretroviral Therapy and Quality of Life of HIV/AIDS Patients in Baoshan, China: A Randomized Controlled Trial. AIDS Res Treat.

[29] Shet A, De CA, Kumarasamy N, Rodrigues R, Rewari BB, Ashorn P, Eriksson B, Diwan V (2014). Effect of mobile telephone reminders on treatment outcome in HIV: evidence from a randomised controlled trial in India. BMJ.

[30] Mutimura E, Addison D, Anastos K, Hoover D, Dusingize JC, Karenzie B, Izimukwiye I, Mutesa L, Nsanzimana S, Nash D, IeDEA Central Africa Collaboration (2015). Trends in and correlates of CD4+ cell count at antiretroviral therapy initiation after changes in national ART guidelines in Rwanda. AIDS.

[31] IeDEA and COHERE Cohort Collaborations (2018). Global Trends in CD4 Cell Count at the Start of Antiretroviral Therapy: Collaborative Study of Treatment Programs. Clin Infect Dis.

[32] Bulage L, Ssewanyana I, Nankabirwa V, Nsubuga F, Kihembo C, Pande G, Ario AR, Matovu JK, Wanyenze RK, Kiyaga C (2017). Factors Associated with Virological Non-suppression among HIV-Positive Patients on Antiretroviral Therapy in Uganda, August 2014-July 2015. BMC Infect Dis.

[33] Oliver Rowntree (2019). Sizing the mobile gender gap. The Mobile Gender Gap Report 2019.

[34] NITA-U (2018). National Information Technology Survey: 2017/18 Report.

[35] Twimukye Adelline (2017). Exploring Attitudes and Perceptions of Patients and Staff towards “Call For Life” System for HIV Antiretroviral Therapy in Uganda: A Qualitative Study. http://saafrica.org/new/wp-content/uploads/2018/07/ICASA-2017-Programme-Book.pdf.

[36] Nabaggala M, Kiragga A, Naggirinya NA, Oseku E, Akirana J, Oyat P, Pattery T, Ratanshi R (2019). OP15 Use Of Digital Health Information Among HIV Populations In Uganda. Int J Technol Assess Health Care.

[37] Musiimenta A, Tumuhimbise W, Mugaba AT, Muzoora C, Armstrong-Hough M, Bangsberg D, Davis JL, Haberer JE (2019). Digital monitoring technologies could enhance tuberculosis medication adherence in Uganda: Mixed methods study. J Clin Tuberc Other Mycobact Dis.

[38] Oseku E, Nabaggala M.S, Naggirinya A.B, Ahumuza T, Musinguzi F, Lamorde M, Asiimwe D, King R, Owarwo N, Laker E, Orama R, Castelnuovo B, Kiragga A, Byonanebye D.M, Parkes-Ratanshi R (2019). Using interactive voice response for PLHIV on art: patient interaction with mHealth. https://conf.helina-online.org/public/2019_Helina_Proceedings_Part1.pdf.

[39] Rodrigues R, Shet A, Antony J, Sidney K, Arumugam K, Krishnamurthy S, D'Souza G, DeCosta A (2012). Supporting adherence to antiretroviral therapy with mobile phone reminders: results from a cohort in South India. PLoS One.

[40] Hermans SM, Elbireer S, Tibakabikoba H, Hoefman BJ, Manabe YC (2017). Text messaging to decrease tuberculosis treatment attrition in TB-HIV coinfection in Uganda. Patient Prefer Adherence.

[41] Haberer JE, Kiwanuka J, Nansera D, Wilson IB, Bangsberg DR (2010). Challenges in using mobile phones for collection of antiretroviral therapy adherence data in a resource-limited setting. AIDS Behav.

