# Supplementary material for: An Interactive Voice Response Software to Improve the Quality of Life of People Living With HIV in Uganda: Randomized Controlled Trial
Source: JMIR Mhealth Uhealth. 2021 Feb 11;9(2):e22229. doi: 10.2196/22229 (PMC7906832; doi:10.2196/22229)
Supplement: Multimedia Appendix 1 [file mhealth_v9i2e22229_app1.docx]

**Multimedia Appendix 1:** Issues identified with TAMA/ Call for Life system during the pilot phase

| **Type of issue** | **Details** | **Mitigation in CFL** |
| --- | --- | --- |
| IVR service providers | To reach patients, two IVR service providers are used; one local and the second international. These two had to be configured to work together. This led to several issues including:  Challenges with configurations leading to calls not closing when the patients hung up. This had a large impact on the cost of calls.  Since most existing mHealth services are text message-based, the local IVR provider had limited capacity to address some of the implementation challenges. This created delays when solving issues that came up and some issues have remained unresolved. Failed pin authentication issue was an issue that affected up to 90% at least once. This negatively affected the patients’ perception of the system and has financial implications as the system must call repeatedly for the pin to work.  There were also inconsistencies in the call logs retrieved from the two IVR providers and the application. This added an extra layer of complexity while reviewing the different logs to verify bills and troubleshooting errors in connection. | Initial step; move all activities to the international service provider. This was expensive.  In 2019, the team built their IVR system, which now means that calls can be generated locally, which has reduced the cost by about two thirds. |
| Internet issues | Since the connection between the system and the IVR relies on the internet connection, the system is affected by the quality of the internet. On some occasions, the local provider’s internet shuts down or is suboptimal leading to poor connectivity. On one occasion fibers were damaged during road works, which caused a breakdown in the internet connection and ultimately the service. | Internet gradually improved in Uganda throughout the study |
| Software issues | There are instances where the Call for Life software has not performed as expected. Some of these issues required reconfigurations from the software developers. For example, there were several errors with the importation of data from ICEA which could only be corrected by adjusting the application itself. | Updated versions of CFL overtime solved these issues |
| Spyware | On one occasion the system shut down because of a denial of service attack (Random brute force attack). | To prevent future attacks, the IT team implemented an IP range limit to ensure that access to the servers is only possible within IDI. |
| Patient challenges | There are some challenges on the level of the patient. Some patients have some challenges responding to prompts because of limited familiarity with their phones. This was especially true for patients with new gadgets and patients who are older. Some patients forget their passwords although they were encouraged to use easy to recall passwords. | Sensitisation of patients during the consent process. Initial & res-set PIN is the same for everyone to ease re-setting in case of blocked PIN |
| Power outages | In case of power outages, it can take 1-2 hours to restart the servers. | This was not particularly common at IDI because there is a backup generator, but it could become a serious issue in more remote settings without power backup facilities. |
| Integration with external databases | IDI has a computerized data management system, ICEA, which was integrated with Call for life. This was challenging because there were several misconfigurations and differences in data types and data labels that made it hard to import data when registering patients. | This integration also meant that some functionalities like visit reminders and the consent flow could not be tested before ‘going live’ with the system. This meant that we had to troubleshoot errors after going live. |
| At the level of the phone network | The system has experienced various issues with congestion at the level of the telecom company network. The exact cause of this is not known but the issue is intermittent and variable; the level of congestion varies between 2% and 80%. The congested calls never get through to the patients. The issue initially became minimal when an additional line for routing calls (E1 line) was introduced but it has continued to recur subsequently.  Service upgrades at the telecom providers have sometimes affected configuration settings with the local IVR provider. This has led to complete service breakdown lasting several days on some occasions. | This improved throughout the study as the stability of the mobile network was greater. In addition, the direct relationship between the study team and Telcos helped, also with local IVRS development. |
| Mechanism for monitoring the system | Since this is a 24-hour service, there is a need to make automated monitoring systems to ensure downtimes are detected as soon as they occur. However, we have had monitor this manually which is time-consuming. | Key staff (2-3) are registered on the system and receive calls daily, thereby monitoring the system |
