# Supplementary material for: An Interactive Voice Response Software to Improve the Quality of Life of People Living With HIV in Uganda: Randomized Controlled Trial
Source: JMIR Mhealth Uhealth. 2021 Feb 11;9(2):e22229. doi: 10.2196/22229 (PMC7906832; doi:10.2196/22229)
Supplement: Multimedia Appendix 2 [file mhealth_v9i2e22229_app2.docx]

**Multimedia Appendix 2:** Version changes and system upgrades of Call for life Uganda

| **CFL Version** | **Release date** | **Release notes** |
| --- | --- | --- |
| 1.0.0 | 07/12/2015 | Initial version |
| 2.0.0 | 20/01/2016 | Alerts notification to emergency phone Health tips delivery via SMS Remove SMS from the preferred way of communication Email domain validation clinicians Redesign treatment -services link Importing past treatments and linking them to medical check-up data Add OTC as a separate message in admin config module Restriction role choice for local admin Error messages stay visible Lab results: date not prepopulated Make password blank Change message for outside the system communication Change labels on the edit clinic page Change the 'street' field on the edit clinic page |
| 3.0.0 | 26/02/2016 | Delete Patient Change search patient by phone A text area for input in Medical History Navigation lab results overview View/edit patient lab result Validation for Lab result page Redesign patient registration flow UI for lab result detail page Enable/disable LRCS config + other configs Nightly sync Local Clinic Patient ID/study code Alerts for imported results |
| 4.0.0 | 10/07/2016 | Patient search on multiple patient ID's LRCS data entry Adherence calculation Underlying alert service Alerts for adherence Adherence graph for every disease/treatment Adherence feedback to the patient Call flow changes for adherence feedback to the patient Health Tip Delivery Service Update Replaying the useful health tips New patient activation flow General + call flow config + alert for the locked pin UI medical history pages (including text field on check-up data page) Script for easy data (config and other data) import after deployment Updated existing and added new report templates |
| 5.0.0 | 06/11/2016 | Registration with consent later Delete Patient completely from CFL Updated call log table to hold all the played files The configuration default setting for Services Page Display treatment changes on the treatment page  Alerts configuration on individual lab result level Configuration default selection services for treatment page  Modification edit lab results for standalone LRCS use Cancellation of scheduled visit(s) Displaying additional patient ID in the alert section String input for lab results Display patient information in the header Registration with Outbound call Add multiple diseases |
| 5.1.0 | 10/01/2017 | New Help page Provide references for uploaded documents Display default search Patient ID Show Estimated Age instead of True Age |
| 5.2.0 | 03/02/2017 | Export/Import of configurations |
| 5.3.0 | 14/03/2017 | Disconnecting the call for "No answer" and "No input" Tracking of actual call time Configure maximum call time Logging of played messages |
| 6.0.0 | 28/04/2017 | Update “Estimated Age” each year Edit past visit date Edit lab result sample date  Highlighting the treatment tab containing error messages The issue with updating the medicine name in the MoTeCH back end. Add missing columns to standard reports |
| 6.0.1 | 02/02/2018 | Specific IDI release with minor updates |
| 6.1.0 | 14/09/2017 | Specific MDACS release with minor updates |
| 7.0.0 | 29/01/2018 | Add column clinic to call detail report Only show open/in progress alerts Multi-select option in Advanced Search (Alerts, Lab Results) Alert for "X consecutive missed visits" Close alerts in bulk Include regimen as a variable in the symptom reporting algorithm Show the patients list Send mails from CFL instead of MoTeCH Remove the encryption from DB fields first name, middle name, surname and Date Of Birth UI Changes to make consent flow more user friendly Option to remove a drug in MoTeCH back-end Create Datamart |
| 7.0.1 | 14/02/2018 | Fix performance issue with maxFetchDepth Consent details on the profile page Remove patient from another clinic from the search result |
| 7.0.2 | 11/05/2018 | Surname and middle name were not populating when importing patients |
| 7.1.0 | 04/06/2018 | Logic to identify the different message parts in one call List Power BI links Fix services page for feedback adherence checkbox |
| 8.0.0 | 24/06/2018 | Migration to new MoTeCH version |
| 9.0.0 | 20/10/2018 | Patient-Caregiver relationship Call Services to Caregivers |
| 9.0.1 | 10/01/2019 | Fix calls which are not placed for a patient |
| 9.0.2 | 14/02/2019 | Fix auto complete |
| 9.1.0 | 04/06/2019 | Upload profile picture Profile Picture in the patient list screen Capture biometric scan during the registration of a patient Fix Creation of a PowerBI link Fix clinic label in French CFL version |
| 9.2.0 | 25/07/2019 | New role for “Call center agent” New role for “Treatment coordinator” Fix duplicate search results when the same caregiver is mapped to different patients. Fix error in treatment page when the user who created/updated the patient treatment details are deleted from the application. Fix the patient identifier search. Fix caregiver search performance Fix SMS for caregivers |
| 9.3.0 | 05/09/2019 | JWT authentication support for call flows module |
