# Supplementary material for: An Interactive Voice Response Software to Improve the Quality of Life of People Living With HIV in Uganda: Randomized Controlled Trial
Source: JMIR Mhealth Uhealth. 2021 Feb 11;9(2):e22229. doi: 10.2196/22229 (PMC7906832; doi:10.2196/22229)
Supplement: Multimedia Appendix 3 [file mhealth_v9i2e22229_app3.docx]

**Multimedia Appendix 3:** Health educational messages (tips) used in the study

| **Category** | **Subcategory** | **Example of a tip in English** | **Number of tips** |
| --- | --- | --- | --- |
| HIV | General | AIDS in full is Acquired Immune Deficiency Syndrome. It is a disease which has no cure that reduces the body's natural defence against illness. It is caused by a virus called HIV. Not everyone with HIV has AIDS. Our bodies are different, some may show signs of AIDS in a shorter time than others. You can slow the progression of HIV to AIDS by getting into care and living positively | 47 |
| ARVs and Adherence | ARVs | ARVs in full are Antiretroviral meaning a combination of drugs that slow HIV from reproducing by protecting CD4 cells in the immune system. ARVs are the main type of treatment for HIV/AIDS. There is no cure for HIV/AIDS, and it can reduce your life length especially if not treated. ARVs can stop people from becoming ill for many years, make symptoms better and prolong life. You MUST follow instructions from your counselor and take the right amount of ARVs at the right time. If you miss a dose, take it as soon as you can and see your counselor. Do not share ARVs with others-if you share you will not get enough doses for them to work properly. Some people believe many things about ARVs that are not true. ARVs do have some side effects but they can be treated. Consult your doctor/ counsellor if you have any questions concerning ARVs. | 21 |
| Positive living | Stigma and discrimination, nutrition | Positive living is a good way of life for anyone. You need to take care of your health and body when you have HIV. People with HIV/AIDS have low immunity, so they catch diseases more easily. Positive living helps boost their immunity and avoid these diseases. Positive living includes avoiding alcohol/smoking, having a balanced diet, drinking fresh clean water, sleeping under a treated mosquito net, taking Septrin daily, using condoms to avoid reinfection and taking ARVs as instructed by a health worker, exercising and getting enough rest, Keeping regular clinic appointments and joining a support group. | 17 |
| General Health | Opportunistic infections & tuberculosis | Opportunistic infections are caused by germs that attack the body because of low immunity. Your immunity can be low due to malnutrition, HIV/AIDs, pregnancy, medication, or chronic disease. People with HIV are more vulnerable to opportunistic infections because of their weakened immune system. The likelihood of opportunistic infections can be reduced by maintaining a good diet and healthy lifestyle, and by taking the medication regularly if you suffer from a chronic illness. Seek diagnosis and treatment from the nearest clinic or trained health worker IMMEDIATELY at the onset of any infection. | 15 |
| Sexuality | Condoms, young adults, family planning, infertility | Sex with No Condom Puts You at Risk of getting HIV/AIDS, STIs and unwanted pregnancy. Prevent transmitting HIV to others or re-infecting yourself by using a condom every time you have sex. Condoms are available and FREE at government health facilities. Free condoms are as good as the ones you buy if the package is sealed and the expiry date is not passed. Heat and strong sun can damage condoms. They should be stored in a cool dry place. Condoms can break if they are poorly stored, expired, damaged, or incorrectly used. Before using a condom check the expiry dates before you unwrap from the package. | 60 |
| Pregnancy and breastfeeding | PMTCT, breastfeeding | You can protect your baby from HIV with exclusive breastfeeding or replacement feeding. Mixing will increase the baby’s chance of getting HIV especially within the first 6 months of life. Exclusive breastfeeding is when a baby is fed with breast milk ONLY. Exclusive replacement feeding means providing a baby with nutrients without using ANY breast milk-special powder milk can give this. HIV positive mothers can safely choose either feeding options if they follow health workers instructions concerning infant feeding. If you decide to breastfeed, you cannot give your baby anything else but breast milk for the first 6 months of their life. When your baby is six months, you can start to introduce other feeds, but you should continue breastfeeding until the baby is one year then wean completely. Return to the health centre to learn how to transition from milk to regular food. | 25 |
| HIV | Discordance, PEP | HIV transmission can be prevented. There are many risk reductions options, and couples need to identify a method or methods that suit them. When you decide on a risk reduction method, it is common for couples to achieve success right away with the help of the counselors who help the couples cope. It is never too late to start practising risk reduction. | 3 |
| HIV | HIV and aging | Just like anybody, getting older means you are more likely to have health problems. HIV accelerates the ageing process and magnifies its effects. Old age increases your chances of getting heart disease, diabetes, cancer, bone weakness, kidney problems, and other diseases. Your health care provider will continue to monitor you for these diseases | 7 |
| HIV | Dating and Relationships | Attraction to the opposite sex is natural but learning how to approach, appreciate and understand someone of the opposite sex is not easy. Its takes time to learn. You might feel pressurized to start dating with your friends, the music or TV programs you watch. Do not give in to such pressure, only get into a relationship with someone you know and trust and only when you are ready. | 4 |
| General health | Diabetes, hypertension, miscarriage, rape cross-generational sex, depression, rational medication use, smoking, allergy | Diabetes is a disease in which blood glucose or sugar levels are too high. Glucose comes from the foods you eat. High blood sugar happens when the body's pancreas no longer produces enough insulin, a substance that helps the glucose get into your cells to give them energy. Diabetes leads to problems with the heart, eyes, kidneys, and nerves. If not treated, diabetes can cause death. See a health worker for diagnosis and treatment if you are worried about having diabetes. | 130 |
