# Supplementary figures and images for: An Interactive Voice Response Software to Improve the Quality of Life of People Living With HIV in Uganda: Randomized Controlled Trial
Source: JMIR Mhealth Uhealth. 2021 Feb 11;9(2):e22229. doi: 10.2196/22229 (PMC7906832; doi:10.2196/22229)

**Multimedia Appendix 4:** Call flow diagram for Call for Life™ Project.

***
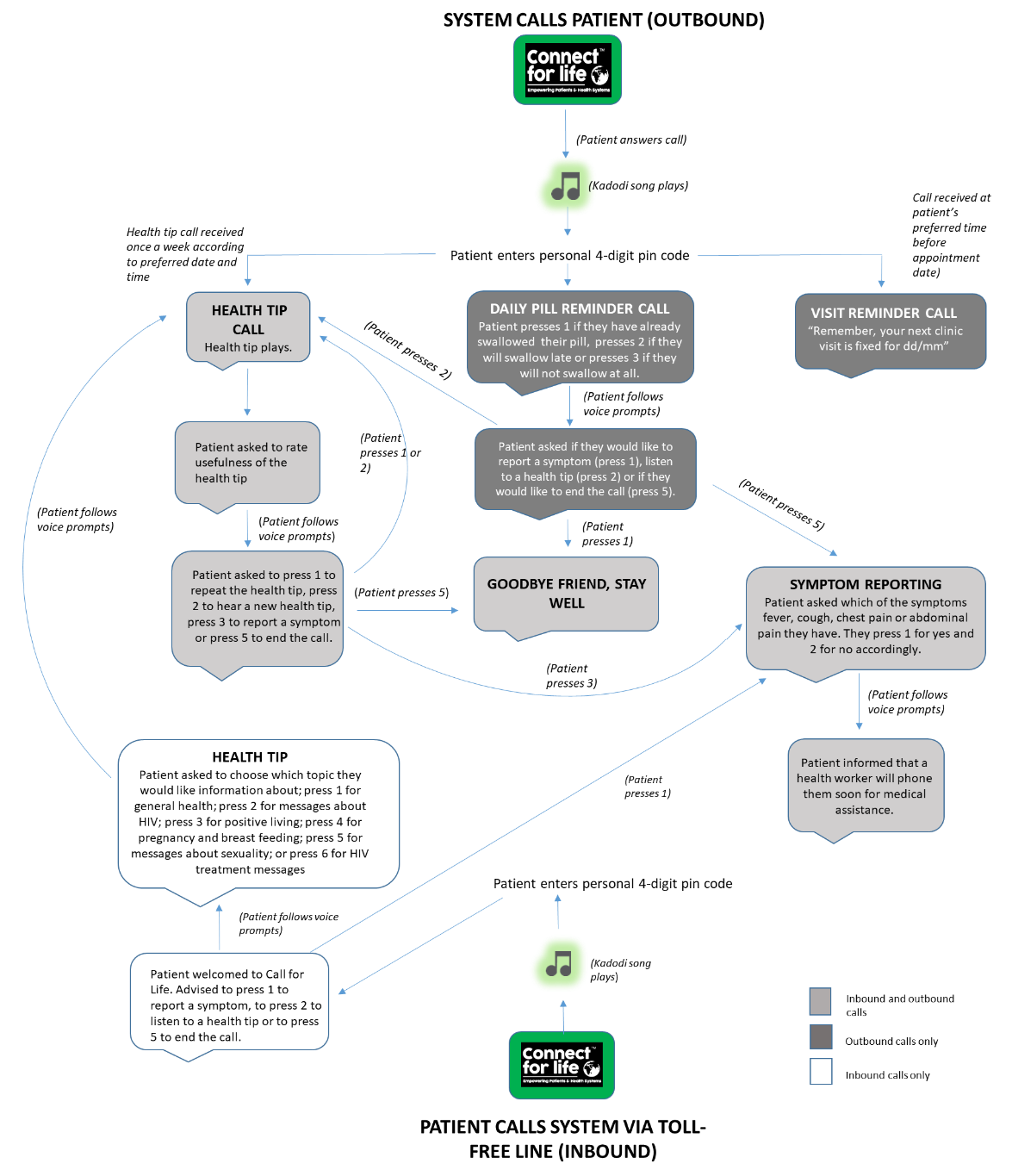
***

Supplement: Multimedia Appendix 4 [file mhealth_v9i2e22229_app4.docx]
