# Supplementary material for: An Interactive Voice Response Software to Improve the Quality of Life of People Living With HIV in Uganda: Randomized Controlled Trial
Source: JMIR Mhealth Uhealth. 2021 Feb 11;9(2):e22229. doi: 10.2196/22229 (PMC7906832; doi:10.2196/22229)
Supplement: Multimedia Appendix 5 [file mhealth_v9i2e22229_app5.pdf]

Improving outcomes in HIV patients using mobile phone based interactive software support

Area: HIV patients receiving ART

Type: Open label randomized controlled trial

List of investigators and co-investigators:

Principle Investigator

Dr. Rosalind Parkes-Ratanshi, MBChB, MRCP, PhD
Infectious Disease Institute
P.O. Box 22418
Kampala, UGANDA
Phone: + 256 41 4307000
Email: rratanshi@idi.co.ug

Co-investigator

Prof Elly Katabira
College of Health Sciences
Makerere University
Kampala, UGANDA

Dr. Barbara Castelnuovo
P.O. Box 22418
Kampala, UGANDA
Phone: + 256 41 4307300
bcastelnuovo@idi.co.ug

Dr. Agnes Bwanika
Infectious Disease Institute
P.O. Box 22418
Kampala, Uganda
Phone: + 256414307000
Email: anaggirinya@idi.co.ug

Dr Noela Owarwo
Infectious Disease Institute
P.O. Box 22418
Kampala, UGANDA
Phone: + 256 41 4307000
Email: nowarwo@idi.co.ug

Agnes N. Kiragga
Infectious Disease Institute
P.O. Box 22418
Kampala, UGANDA
Phone: + 256 41 4307000
Email: akiragga@idi.co.ug

Makerere University School of Public Health • Higher Degrees, Research and Ethics Committee •
Valid Thru:
03 JUL 2020

Eva Laker
Infectious Disease Institute
P.O. Box 22418
Kampala, UGANDA
Phone: + 256 41 4307000
Email: elaker@idi.co.ug

Dr. Mohammed Lamorde
Infectious Disease Institute
P.O. Box 22418
Kampala, UGANDA
Phone: + 256 41 4307000
Email; mlamorde@idi.co.ug

Dr. Namagala Elizabeth
Programme Officer ART
STD/AIDS Control Programme
Ministry of Health
P.o Box 7272 Kampala
Tel: +256772490150
Email: namagalae@yahoo.com

Makerere University School of Public Health • Higher Degrees, Research and Ethics Committee •
Valid Thru:
03 JUL 2020

Document History

Document	Version Date	Summary of Changes
Amendment X	*DD Month YYYY*	
Amendment 2	*16 April 2018*	• *Schedule of follow-up updated: Baseline, 6 Months, 12 Months, 18 Months, 24 Months*<br>• *Control arm extended to 24 months for a sub-set of patients*<br>• *Willingness questionnaire at 18 & 24 months added*<br>• *DSMB member composition*<br>• *Statistical analysis*
Amendment 1	*16 11 2016*	*Subject selection: Added Most at risk population (MARPs)*<br>*Case report forms changed to Data fax forms. Detail on Source documents and Datafax system have been added*<br>*Baseline socio-economic & medical evaluation form updated to capture cost analysis*<br>*Follow-up socio-economic & medical evaluation form added*<br>*Focus group discussion consents added for the qualitative sub- study*<br>*Clarification on interim analysis and inclusion of a DSMB was added.*<br>*Added a new investigator*
Original protocol	17 11 15	N/A

Makerere University School of Public Health • Higher Degrees, Research and Ethics Committee •
Valid Thru:
03 JUL 2020

PROTOCOL SUMMARY

Abstract

Current estimates point towards a huge increase in the number of people that are eligible to start ART in Uganda and globally. As many of the newly eligible patients are largely asymptomatic, there are concerns about adherence and retention of these individuals and especially those starting ART with a higher CD4 counts. Urgent information is required to plan for implementation of most recent WHO and National guidelines in the most cost effective manner as well as maximizing retention of HIV positive individuals in care and achieving virological suppression.

We plan to undertake research designed to see if we can optimize adherence, virological outcomes and HIV knowledge, in order to give an overall increased quality of life in vulnerable populations starting or established on ART in Kampala, Uganda. We will test implementation of an open source software-based tool to send text messages and to give access to an interactive voice response system to support self-monitoring and provide health information using patients' mobile phones.

We aim to undertake an open labeled randomised trial at two sites: the IDI which is an urban center of excellence in HIV care, and Kasangati Health Center, which is a peri-urban public health care facility. The project aims to enroll patients starting ART, already established on first line ART or switching to second line ART. We will include special populations including pregnant women, discordant couples and young people. The estimated length of the project is 30 months. The technology to be evaluated in this study is based on CONNECT FOR LIFE™ m-health technology (CFL2015.01 or higher). CFL2015.01 provides text messages or Interactive Voice Response (IVR) functionalities, a technology that allows a computer to interact with humans through the use of voice and tones input via keypad, including pill reminders, clinic visit reminders, health tips and functionality to support symptom reporting.

The Primary Objective of this study is to determine the effect of the CFL2015.01 tool on quality of life of HIV patients receiving care at IDI and Kasangati HCIV. At the start of the intervention, all patients will undergo quality of life assessment, which will be repeated at months 6, 12, and 24 months. The scores will be compared to assess the effect of the tool on quality of life. The Secondary Objectives are virological outcomes baseline, 6, 12 months and 24 months, retention in care, aversion of early treatment failure, disease knowledge, clinic attendance and cost analysis.

Makerere University School of Public Health
Valid Thru:
03 JUL 2020
Higher Degrees, Research and Ethics Committee

LIST OF ABBREVIATIONS AND DEFINITIONS OF TERMS

**AE**	Adverse events
**CRF**	Case report form
**CYP**	cytochrome P450
**HIV**	Human Immunodeficiency Virus
**IDI**	Infectious Diseases Institute
**IRB**	Institutional Review Board
**UNCST**	Uganda National Council of Science and Technology
**LTFU**	Lost To Follow Up
**MU-JHU**	Makerere University – John's Hopkins University Core Laboratory
**NRTI**	Nucleoside reverse transcriptase inhibitor
**NNRTI**	Non-nucleoside reverse transcriptase inhibitor
**SOC**	Standard of care
**SAE**	Serious Adverse Event
**TAMA**	Treatment Advise by Mobile Alerts
**C4L**	Call for Life Uganda™
**CFL2015.01**	Connect for Life™ technology 2015 Uganda version
**DSMB**	Data Safety & Monitoring Board

Makerere University School of Public Health
Valid Thru:
03 JUL 2020
Higher Degrees, Research and Ethics Committee

TABLE OF CONTENTS

LIST OF ABBREVIATIONS AND DEFINITIONS OF TERMS 6
1. INTRODUCTION 10
    1.1. Disease Setting/Patient Population 10
    1.2. Background and Rationale 10
    1.3. Interactive Voice Response System 12
2. STUDY OBJECTIVES AND ENDPOINTS 14
    2.1. Objectives 14
3. STUDY DESIGN 16
4. SUBJECT SELECTION 17
    4.1. Inclusion Criteria 17
    4.2. Exclusion Criteria 17
5. DESCRIPTION OF INTERVENTION 18
6. STUDY PROCEDURES 19
    6.1. Screening 19
    6.2. Study Period 19
    6.3. Follow-up Visit 20
    6.4. Subject Withdrawal 21
7. ASSESSMENTS 25
    7.1. Safety 25
        7.1.1. Unanticipated Problems 25
    7.2. Pregnancy Testing 25
    7.3. Post-Recruitment Illness 25
8. ADVERSE EVENT REPORTING 25
    8.1. Adverse Events 25
    8.2. Serious Adverse Events 26
    8.3. Severity Assessment 26
    8.4. Causality Assessment 26
    8.5. Reporting Requirements 26
9. DATA ANALYSIS/STATISTICAL METHODS 26
    9.1. Sample Size Determination 26
    9.2. Analysis of Endpoints 27
        9.2.1. Analysis of Primary Endpoint 27

Makerere University School of Public Health • Higher Degrees, Research and Ethics Committee •
Valid Thru:
03 JUL 2020

9.2.2. Analysis of Secondary Endpoints 27
9.3. Interim Analysis 30
10. QUALITY CONTROL AND QUALITY ASSURANCE 30
11. DATA HANDLING/RECORD RETENTION 30
11.1. Case Report Forms (CRF)/Electronic Data Record 30
11.2. Record Retention 33
11.3. Confidentiality 33
12. ETHICS 34
12.1. Institutional Review Board (IRB) 34
12.2. Ethical Conduct of the Study 34
12.3. Subject Information and Consent 34
12.4. Data Privacy 34
13. DEFINITION OF END OF TRIAL 35
14. PUBLICATION OF STUDY RESULTS 35
15. CAPACITY BUILDING 35
16. FUNDING 36
17. REFERENCES 38

Makerere University School of Public Health • Higher Degrees, Research and Ethics Committee •
Valid Thru:
03 JUL 2020

1. INTRODUCTION

Despite the large scale up of HIV treatment to over 550,000 patients receiving antiretroviral therapy (ART) in Uganda, there are an estimated 60% of eligible patients who are not receiving treatment (1, 2). This represents a substantial gap in services, and closing this gap will have major implications for health services in Uganda. The situation is similar elsewhere in Sub-Saharan(3). Under the most recent 2013 WHO HIV treatment guidelines, which recommend treatment at higher CD4 counts than previously, many of the eligible patients are largely asymptomatic. This raises concerns about adherence and retention of these individuals initiating treatment with a higher CD4 counts. Urgent information is required to plan for continued scale up of HIV treatment in Uganda in the most cost effective manner as well as maximizing retention of HIV positive individuals in care and achieving virological suppression(4). In addition patients belonging to vulnerable groups (or key populations), such as discordant couples, pregnant women, young adults and those on second line treatment are more likely to have issues with adherence and therefore these patients may require additional support for their HIV care(5).

ART adherence, with consequent reduction in viral load and increase in CD4 count positively influences quality of life in those living with HIV(6). Attendance at clinic appointments is a good predictor of good outcomes in HIV patients(7). Support mechanisms and HIV knowledge also have been shown to have an effect on quality of life(8, 9). mHealth technologies offer us the potential to address many of these aspects in one system, and the aim of this trial is to test a system developed to improve on all of these outcomes.

Disease Setting/Patient Population
We plan to study patients starting ART, or belonging to special populations who are already on ART including pregnant women, discordant couples and young people or switching to second line ART at 2 clinics; IDI, Kampala and Kasangati HCIV. The estimated length of the project is 30 months.

1.1. Background and Rationale

The estimates of eligible patients in Uganda and globally point towards a huge increase in numbers of people who are now eligible to start ART(3) under the new 2003 WHO guidelines compared to previously, which has major implications for health services in Sub-Saharan. Urgent research is required to assist with implementation of these guidelines in the most cost effective manner(4).

One of the major changes in delivery of HIV care is that patients are starting to take ART at higher CD4 counts, often when they are feeling well. We know from resource rich settings that implementing treatment at high CD4 counts is not straightforward. In the USA, people have started on ART at high CD4 counts (>500) for a number of years. However, at present only 77% of those who are on ART in the USA have

Makerere University School of Public Health
Valid
03 JUL 2020
Higher Degrees, Research and Ethics Committee

managed to achieve an undetectable viral load load (giving an overall 28% of all those infected with HIV achieving virological suppression).

In sub-Saharan Africa a meta-analysis of HIV patients showed that those with high CD4 counts were more likely to be lost to follow up (25% with CD4 count <350 vs. 54% with CD4 counts >350) ($P < 0.0001$)(10). This suggests that these patients at risk of poor outcomes as in Uganda, ART eligible patients lost to follow up before starting ART have a 7.7% mortality(11). For those who do start on ART in sub-Saharan Africa 76% have an undetectable viral load at one year and 67% at 2 years (12). ART adherence in sub-Saharan Africa is a complex issue with multiple contributing factors(13). However, large programmatic data on outcomes in patients starting at high CD4 counts in Sub-Saharan Africa are not currently available. Experts in mathematical models from South Africa have highlighted that optimizing adherence in those starting ART at high CD4 counts as a priority for research (14).

Adherence in Key Populations

Due to their increased vulnerability and risk of transmission key populations (such as adolescents, pregnant women and discordant couples) require tailored responses and assistance with managing their HIV, as recognized in the 2014 WHO guidelines for key populations(5). In particular there is emerging evidence that suggests it may be difficult to persuade HIV positive partners in discordant couples to start ART, and this may be more challenging at higher CD4 counts. In Kenya only 63% of ART eligible partners in discordant couples started ART by 1 year into care (10). At the Infectious Diseases Institute (IDI) in Kampala, we have found that HIV positive partners in discordant couples are less likely to start ART if their CD4 counts are greater than 350 cells/uL as compared to those with low CD4 count(15).

There are also concerns regarding poor uptake of PMTCT services of any kind, despite widespread availability in Sub-Saharan Africa. A review of 40 studies in Sub-Saharan Africa cited poor knowledge of HIV/ART/vertical transmission, lower maternal educational level and psychological issues following HIV diagnosis as some of the key individual barriers to access PMTCT, whereas poor staff-client interactions, staff shortages, service accessibility were some of the clinical constraints(16). Even women who access ART for during pregnancy for their own health (are at a high risk of stopping ART or becoming LTFUP (numbers) after their pregnancy. (17)

Due to adherence concerns the WHO guidelines emphasize the need of strong systems to link patients to care and to enhance adherence in those starting ART. The guidelines recommend **"research priorities include assessing the incidence of severe adverse events as a result of increased exposure to ART and assessing ART acceptability, uptake, adherence and long-term retention in care for people who initiate ART at higher CD4 counts"(2).**

Mobile Phone alerts for adherence and appointment reminders

Data from randomized controlled trials (RCT) and cohort analyses show improved virological outcomes and/or adherence with text messaging alerts (18-24). A large

Makerere University School of Public Health • Higher Degrees, Research and Ethics Committee
03 JUL 2020

RCT in Kenya trial compared weekly text messages versus standard of care. Text messaging was associated with a lower risk of non-adherence at 12 months (RR 0.77, 95% CI 0.63 to 0.93) and with the non-occurrence of virological failure at 12 months (RR 0.83, 95% CI 0.69 to 0.99) (19). Another trial in Kenya compared short daily, long daily, short weekly and long weekly messages against standard care. Patients receiving weekly text-messages of any length were at lower risk of non-adherence at 48 weeks than were patients receiving daily messages of any length (RR 0.79, 95% CI 0.64 to 0.99). Other studies have looked at 2 way communication via text message or voice activated mobile phone support for improving adherence (21). In South India voice activated messaging system compared to a simple text message was preferable for patients(25). In Cameroon an intervention with 2 way communication was utilized mainly for logistical (n = 21, 13.6%), medical (n = 20, 12.9%) and financial (n = 11, 7.1%) support (26). Currently there is also one ongoing study of using text messages to retain patients not yet on ART in care. (27)

We found one study using text messages in women accessing PMTCT services; this was a study in which pregnant women could access information via a text message service. Participants on average sent 16 messages to the study team per week, 50% requesting medical information, the remainder for social/psychological support(28). We were unable to find any studies looking at text message interventions in improving adherence in discordant couples.

A 2013 Cochrane review showed that mobile phone text messaging reminders increase attendance at healthcare appointments compared to no reminders and postal reminders, and have the same impact on attendance as phone call reminders. Two studies reported that the costs per attendance of mobile phone text message reminders are less than phone call reminders[31].

1.2. The effect of HIV knowledge on adherence and quality of life

There is data to suggest that patients with higher HIV knowledge are twice as likely to keep their appointments as those who are less knowledgeable. In combination with relationship with provider, knowledge predicted increased CD4 count and increased the chance of achieving an undetectable VL by almost five times (7). Lack of knowledge of correct dosing and time to take drugs was associated with adherence issues in those starting ART(29). HIV knowledge has also been shown to be associated with improved quality of life(8).

1.3. Interactive Voice Response System

The technology to be evaluated in this study is based on CONNECT FOR LIFE™ m-health technology. It has been developed as a community health information technology platform for health initiatives to assist patients in developing countries. As a mobile communication platform it interacts with patients using basic mobile phone technology and with healthcare providers through a web-based interface. It provides text message or Interactive Voice Response (IVR) functionalities, a

Makerere University School of Public Health • Higher Degrees, Research and Ethics Committee • Valid Through
03 JUL 2020

technology that allows a computer to interact with humans through the use of voice and tones input via keypad, including pill reminders, clinic visit reminders, health tips and functionality to support symptom reporting.

This IVR software was first piloted by Janssen and the Grameen Foundation in India, and was called TAMA (Treatment advice by Mobile Alerts) (http://motechsuite.org/index.php/implementations) to support HIV patients in project UNITE in India. Early qualitative work suggested that the TAMA software was usable and viable in the real life settings of PLHA and it had many desirable effects on their treatment adherence[31]. The results of Project UNITE are currently in the process of publication.

The CONNECT FOR LIFE™ software is designed to help patients in a number of ways; to improve adherence, to remind about appointments, to provide psychological support by having a way of contacting the clinic 24 hours a day, to increase knowledge of HIV and health related conditions. Therefore, we wish to understand the overall effect of the tool on the patients. Measures of quality of life (QOL) are a composite of many factors, including varied domains of functioning and wellbeing which comprise physical and mental health(9). QoL scores are well correlated with response to HIV treatment in those starting ART(30), but are also useful in measuring response in chronic HIV(31). In addition to medical and symptom effects on quality of life, informational support has been shown to have a positive influence on QOL(8). Therefore, in this trial our primary outcome will be a comparision of QOL in those receiving or not receiving support from CONNECT FOR LIFE™.

The WHO has provided guidance surrounding use of text messages for adherence; **"Mobile phone text messages could be considered as a reminder tool for promoting adherence to ART as part of a package of adherence interventions"(32)**. After discussion with MoH IDI plans undertake a study of the CONNECT FOR LIFE™ m-health technology (CFL2015.01 or higher) within a program named Call for Life Uganda™ which inludes content developed for HIV patients in Uganda uploaded to the CONNECT FOR LIFE™ technology and the implementation of this in clinic. This study will help to gather additional information on outcomes in those in the Call for Life Uganda™ programme , as well as cost analyses and information on operational issues.

Makerere University School of Public Health • Higher Degrees, Research and Ethics Committee •
Valid Thru:
03 JUL 2020

2. STUDY OBJECTIVES AND ENDPOINTS

2.1. Objectives

Primary Objective

To determine the effect of Call for Life Uganda™ on physicial quality of life of HIV patients receiving care at IDI and Kasangati HCIV.

Secondary objectives

1. To determine the effect of Call for Life Uganda™ on mental quality of life of HIV patients receiving care at IDI and Kasangati HCIV.
2. To determine the effect of Call for Life Uganda™ on virological outcomes and aversion of early treatment failure at 6, 12, 18 and 24 months of using the tool
3. To determine effect of use of Call for Life Uganda™ on retention in care at IDI and Kasangati
4. To compare the knowledge about HIV, ART and associated health conditions at baseline, 6, 12, 18 and 24 months in patients using the Call for Life Uganda™ tool
5. To determine the effect of Call for Life Uganda™ use on adoption of risk reduction behaviour
6. To assess patient perceptions and attitudes towards the Call for Life Uganda™ tool including but not limited to a) passive refusal to engage with Call for Life Uganda™ calls, b) qualitative responses around which features are most useful, c) value addition and preparedness of patients to pay for service for sustainability
7. To carry out a cost analysis that can inform 1) the cost of Call for Life Uganda™ intervention in the IDI and Kasangati HCIV populations 2) the cost of rolling out Call for Life Uganda™ for special populations if Call for Life Uganda™ is rolled-out nationwide 3) the estimated cost of rolling out Call for Life Uganda™ in all HIV patients stating ART nationwide
8. To determine the effect of Call for Life Uganda™ on attendance on scheduled appointment date (+/- 2 working days)

Makerere University School of Public Health • Higher Degrees, Research and Ethics Committee
Valid Thru:
03 JUL 2020

Primary Endpoint

1. Change in quality of life physical health score in those accessing Call for Life Uganda™ between baseline, 6 , 12, and 24 months of Call for Life Uganda™

use, and comparison to those with no access to Call for Life Uganda™ at 6, and 12 months

Secondary Endpoints

1. Change in quality of life mental health score in those accessing Call for Life Uganda™ between baseline, 6 and 12 months of Call for Life Uganda™ use, and comparison to those with no access to Call for Life Uganda™ at 6 and 12 months

2. Proportion of patients with viral load > 50 copies/ml at baseline, 6, and 12, months among patients using the Call for Life Uganda™ tool compared to those with no access to Call for Life Uganda™

3. Proportion of patients lost to follow-up (LTFUP), where LTFUP is defined as no having a clinic encounter for > 3 months from the last attendance in each arm

4. Knowledge score about HIV, ART and associated health conditions at baseline and after 6, 12, 18 and 24 months of Call for Life Uganda™ use

5. Proportion using condom and number of sexual partners at baseline and after 6, 12, 18 and 24 months among patients who received Call for Life Uganda™.

6. Proportion of patients who accept to engage with tool more than one time (disaggregated by patient population)

7. Qualitative assessment of patient satisfaction with the Call for Life Uganda™ platform

8. Total costs of Call for Life Uganda™ at IDI, compared those accessing care at IDI with no access to Call for Life™ in cost of care for IDI, the patient and the overall healthcare system. (number of clinic visits/ time to switch to second line treatment, travel costs, days out of work)

9. Qualitative assessment of pattern of calls and texts requested by patients using Call for Life Uganda™ over time. I.e. analysis of real use of the system by patient preference over time

In evaluating mHealth tools, new guidance was published in 2016 on how to report and assess mHealth interventions (mERA). These include access of individual participants and cost assessment which are represented above (33).

2.2 Study Hypothesis

We hypothesize that:

Patients with access to Call for Life Uganda™ will have a better quality of life after 12 months on Call for Life Uganda™ compared to those not on Call for Life Uganda™

Makerere University School of Public Health • Higher Degrees, Research and Ethics Committee •
Valid Thru:
03 JUL 2020

3. STUDY DESIGN

This is a randomised controlled trial at two sites: the IDI which is a center of excellence in HIV care, and Kasangati Health Center, which is a public health care facility. There will be two arms, randomized in a 1:1 pattern. At the start of the intervention, all patients will undergo quality of life assessment, which will be repeated at months 6, 12 and 24. The scores will be compared to assess the effect of the tool.

Following the first 12 months of randomized trial, Call for Life Uganda™ will be offered to all patients on the control arm (if no harm is observed at interim analysis). Observational data will be collected for all parameters for 24 months after trial start.

Makerere University School of Public Health • Higher Degrees, Research and Ethics Committee •
Valid Thru:
03 JUL 2020

FIGURE 1 – C4L STUDY SCHEMA

4. SUBJECT SELECTION

This study will include patients starting ART at IDI and Kasangati as well as those on ART in the following groups; patients in a sero-discordant relationship(most at risk populations, defined by the nature of their work), pregnant mothers, adolescents and those switching or on second line treatment who agree to receive and use the Call for Life Uganda™ tool, and to be followed up for 30 months.

4.1. Inclusion Criteria

Subjects must meet all of the following inclusion criteria to be eligible for enrollment into the study:

1. Evidence of a personally signed and dated informed consent document indicating that the subject (or a legal representative) has been informed of all pertinent aspects of the study.

2. Patients who are willing and able to comply with scheduled visits, treatment plan, laboratory tests, and other study procedures.

3. Patients having the ability to use basic cell phone functions (e.g., being able to make and receive phone calls).

4. Patients with access to a mobile phone (need to be able to answer the phone at a predetermined time slot and to be able to call the clinic if they are sick)

5. Patients who are able to understand Luganda or Runyankole or English (as these are the languages of the overwhelming majority of patients at IDI and Kasangati and so will be used for the trial. If any other language has more than 30 patients who require it, the tool will be adapted to include this new language before the roll out of the tool to the entire clinic population)

4.2. Exclusion Criteria

1. Patient whose clinical condition interferes with appropriate use of cell phone (e.g., deafness, severe cognitive impairment)

2. Patients < 15 years unless emancipated minors as defined by Ugandan National Research Ethics Committee guidelines.

3. Patients who are enrolled in an interventional study at IDI

4. Patients who are not receiving standard first and second line treatment

Makerere University School of Public Health
Valid Thru:
03 JUL 2020
Higher Degrees, Research and Ethics Committee

5. Patients who are critically ill.

5. DESCRIPTION OF INTERVENTION

Call for Life Uganda™

Janssen Global Public Health Research and Development, in close collaboration with the Infectious Disease Institute Kampala (IDI), has developed Call for Life Uganda™ tailored to the needs of PLHIV in Uganda. Call for Life Uganda™ is based on the CONNECT FOR LIFE™ technology (CFL2015.01 or higher version) and the MOTECH platform, an open source platform developed by Grameen Foundation and the University of Southern Maine with financial support from the Bill and Melinda Gates Foundation, and was released under the terms of the MOTECH open source license agreement.

All patients consenting will;

1) At enrolment receive detailed information from a nurse/counsellor on the Call for Life Uganda™ system and how it operates
2) Receive calls or text messages from Call for Life Uganda™ will include recording of self-reported adherence (soliciting active feedback via keypad) and symptom management triggering alerts for the clinic.
3) Participants with an undetectable viral load (after discussion with their counselors) will have the option chose the frequency of their calls, between daily to weekly to a minimum of an appointment reminder (every 1-2months) only.
4) Participants with a detectable viral load at any time will receive daily calls or text messages. They will only be able to decrease the frequency of calls when their VL becomes undetectable and on discussion with their counsellor
5) In addition all patients will receive
    a. an appointment reminder before their appointment (exact timing will be patients choice).
    b. health tips on HIV, ART and related conditions each week (or repeat of the old one if they wish).
    c. have the opportunity to report symptoms to the clinic at any point in time, which send an alert to the clinic (with a call back from clinic staff).

At the end of the study, the study team and a trial steering group made of 3 independent member (a statistician, a mental health specialist and a clinical researcher) will review the data, and if there is no harm seen all of those on the control arm will be offered Call for Life Lite, which provides weekly adherence calls and appointment reminders only, as we plan to offer Call for Life Lite to all patients in the clinic by this time. Those on the full Call for Life arm will continue on this arm. If a positive benefit is seen full Call for Life will be offered to all in the clinic (as long as there are sufficient funding to allow for the phone connection aspect of the tool).

Makerere University School of Public Health • Higher Degrees, Research and Ethics Committee •
Valid Thru:
03 JUL 2020

6. STUDY PROCEDURES

6.1. Screening

The subjects will be screened and enrolled over a period of 6 months

These are the screening procedures

- All consecutive eligible patients will be approached for participation in the study.
- For patients interested in participating the inclusion and exclusion criteria will be ascertained.
- The patients will receive information about the study by the study nurse or counselor
- Patients who agree to participate in the study will sign the informed consent form.

6.2. Study Period

The total duration of the study will be 36 months; follow up up to 24 months

The table below shows the summary of the procedures for the **patients receiving the Call for Life Uganda™ tool**

Study procedures	Baseline	Month 6	Year 1	18 months	Year 2
Screening	√				
Informed Consent	√				
Quality of Life assessment	√	√	√	√	√
Demographic and socio-economic questionnaire	√			√	√
Laboratory					
Viral load	√	√	√	√	√
Resistance testing	*	**	**		**
Plasma storage	√	√	√		√
Cost questionnaire	√	√	√	√	√
Knowledge questionnaire	√	√	√	√	√
Qualitative assessment	√***	√	√	√	√
Sexual behavior questionnaire	√	√	√	√	√
Willingness Questionnaire				√	√

** : if subsequent viral failure*
*** : if VL> 1,000 copies/ml*
***: *Obtain Focus group discussion (FGD) consent or in depth interview (IDIv) consent*

Makerere University School of Public Health • Higher Degrees, Research and Ethics Committee • Valid Thru: 03 JUL 2020

The qualitative analysis will ongoing throughout the study and the FGD/IDIv will continue through all time points. Study participants will be approached to join specific FGD/IDI and so not all study participants will take part in a qualitative assessment at all-time points.
Note: CD4 count test will be performed as per SOC every 6 months for the first year and then yearly. Pregnancy test for the non-pregnant women will be performed if indicated as part of SOC

6.3. Follow-up Visit

The participants will come to the clinic monthly or 2 monthly to pick up their ART and concomitant medications (non-study visits).

The participants will come for their study follow up visits every 6 months

Every 6 months (Month 6, 12, 18, and 24)
The patients will be assessed by the study staff.

- The study doctor will collect General Medical History and perform Physical Examination and enter it in an electronic CRF.
- The counselor will collect the Quality of life and Knowledge questionnaires and enter them in an electronic CRF.

At baseline, 6, 12, 18 and 24 months the following samples will be collected;

- Viral load
- Storage plasma (1 aliquot) serum (3 aliquots)
- Month 18 or 24 Assess willingness to pay for the adherence services after the end of the study in a quantitative analysis
- Assess willingness to have daily calls versus weekly calls

In case of death

- Verbal autopsy by phone or by visiting the home of the deceased
- Review of the clinical information in the clinic and study file to identify a presumptive cause of death

Additional procedures
Resistance testing: It will be done on stored samples

- Baseline. Resistance testing will be carried out if subsequent viral failure in order to investigate transmitted resistant virus
- Biological samples will also be stored at 6, 12, 18, 24 months and resistance testing carried out in case of viral load >1,000 copies/ml

On stored sample
Additional procedures on stored samples may be conducted during the course of the study if deemed to be relevant to improve clinical care will be made available to the clinicians and the study participants.

Makerere University School of Public Health • Higher Degrees, Research and Ethics Committee •
Valid Thru:
03 JUL 2020

6.4. Subject Withdrawal

Subjects may withdraw from the study at any time at their own request (with draw of consent), death, lost to follow-up or they may be withdrawn at any time at the discretion of the investigator for safety or behavioral reasons, or the inability of the subject to comply with the protocol required schedule of study visits or procedures.

If a subject does not return for a scheduled visit, every effort should be made to contact the subject. In any circumstance, every effort should be made to document subject outcome, if possible. The investigator should inquire about the reason for withdrawal and follow-up with the subject regarding any unresolved adverse events (AEs).

If the subject withdraws from the study, and also withdraws consent for disclosure of future information, no further evaluations should be performed, and no additional data should be collected. IDI may retain and continue to use any data collected before such withdrawal of consent.

Loss to follow up in this study is defined as failure to attend for a clinic appointment for three consecutive months after a scheduled appointment. At the end of each visit the participant will be given an appointment for their next scheduled visit. Participants called by the study staff if they do not attend the clinic on the scheduled date. If a participant does not attend for three consecutive months, they will be considered as lost to follow up at clinic thus an early withdraw form will be completed. If they show up after this time period, they will still be followed and will carry on with the next (from that point forward) scheduled visit.

C4L EXTENDED FOLLOW UP SUB STUDY (ADDED IN PROTOCOL 5.2)

Due to issues with the software in late 2017/ early 2018 it was determined that by 12 month follow up not all patients would have received the full dose of the Call for Life intervention. Therefore, a sub-study has been introduced to evaluate a longer control follow up (24 months) for a sub-set of patients who had not transferred to Call for Life Lite from the control arm. This subset includes 170 patients who have not yet transferred from control arm to Call for Life™ lite.

6.5. Objectives

Makerere University School of Public Health Higher Degrees, Research and Ethics Committee
Valid Thru: 03 JUL 2020

Primary Objective of extended follow up sub-study

To determine the effect of Call for Life Uganda™ on physical quality of life of HIV patients receiving care at IDI and Kasangati HCIV.

Secondary objectives

1. To determine the effect of Call for Life Uganda™ on mental quality of life of HIV patients receiving care at IDI and Kasangati HCIV.

2. To determine the effect of Call for Life Uganda™ on virological outcomes and aversion of early treatment failure at 18 and 24 months of using the tool

3. To compare the knowledge about HIV, ART and associated health conditions at baseline, 6, 12, 18 and 24 months in patients using the Call for Life Uganda™ tool

4. To assess patient perceptions and attitudes towards the Call for Life Uganda™ tool including but not limited to a) passive refusal to engage with Call for Life Uganda™ calls, b) qualitative responses around which features are most useful, c) value addition and preparedness of patients to pay for service for sustainability

5. To carry out a cost analysis that can inform 1) the cost of Call for Life Uganda™ intervention in the IDI and Kasangati HCIV populations 2) the cost of rolling out Call for Life Uganda™ for special populations if Call for Life Uganda™ is rolled-out nationwide 3) the estimated cost of rolling out Call for Life Uganda™ in all HIV patients stating ART nationwide

6. To determine the effect of Call for Life Uganda™ on attendance on scheduled appointment date (+/- 2 working days)

7. Assess willingness to get daily versus weekly Call for life tool

Primary Endpoint

Change in quality of life physical health score in those accessing Call for Life Uganda™ between baseline, 18 and 24 months of Call for Life Uganda™ use, and comparison to those with no access to Call for Life Uganda™ at 18 and 24 months

Secondary Endpoints

1. Change in quality of life mental health score in those accessing Call for Life Uganda™ between baseline, 18 and 24 months of Call for Life Uganda™ use, and comparison to those with no access to Call for Life Uganda™ at 18 and 24 months

2. Proportion of patients with viral load > 50 copies/ml at 18, and 24 months among patients using the Call for Life Uganda™ tool compared to those with no access to Call for Life Uganda™

3. Proportion of patients lost to follow-up (LTFUP), where LTFUP is defined as no having a clinic encounter for > 3 months from the last attendance in each arm

Makerere University School of Public Health • Higher Degrees, Research and Ethics Committee
03 JUL 2020

4. Knowledge score about HIV, ART and associated health conditions at baseline and after 18 and 24 months of Call for Life Uganda™ use

5. Proportion using condom and number of sexual partners at baseline and after 18 and 24 months among patients who received Call for Life Uganda™ .

Sub - Study Hypothesis

We hypothesize that:

Patients with access to Call for Life Uganda™ will have a better quality of life after 18 and 24 months on Call for Life Uganda™ compared to those not on Call for Life Uganda™

Makerere University School of Public Health • Higher Degrees, Research and Ethics Committee •
Valid Thru:
03 JUL 2020

FIGURE 2 – CALL FOR LIFE FOLLOW UP SUB STUDY

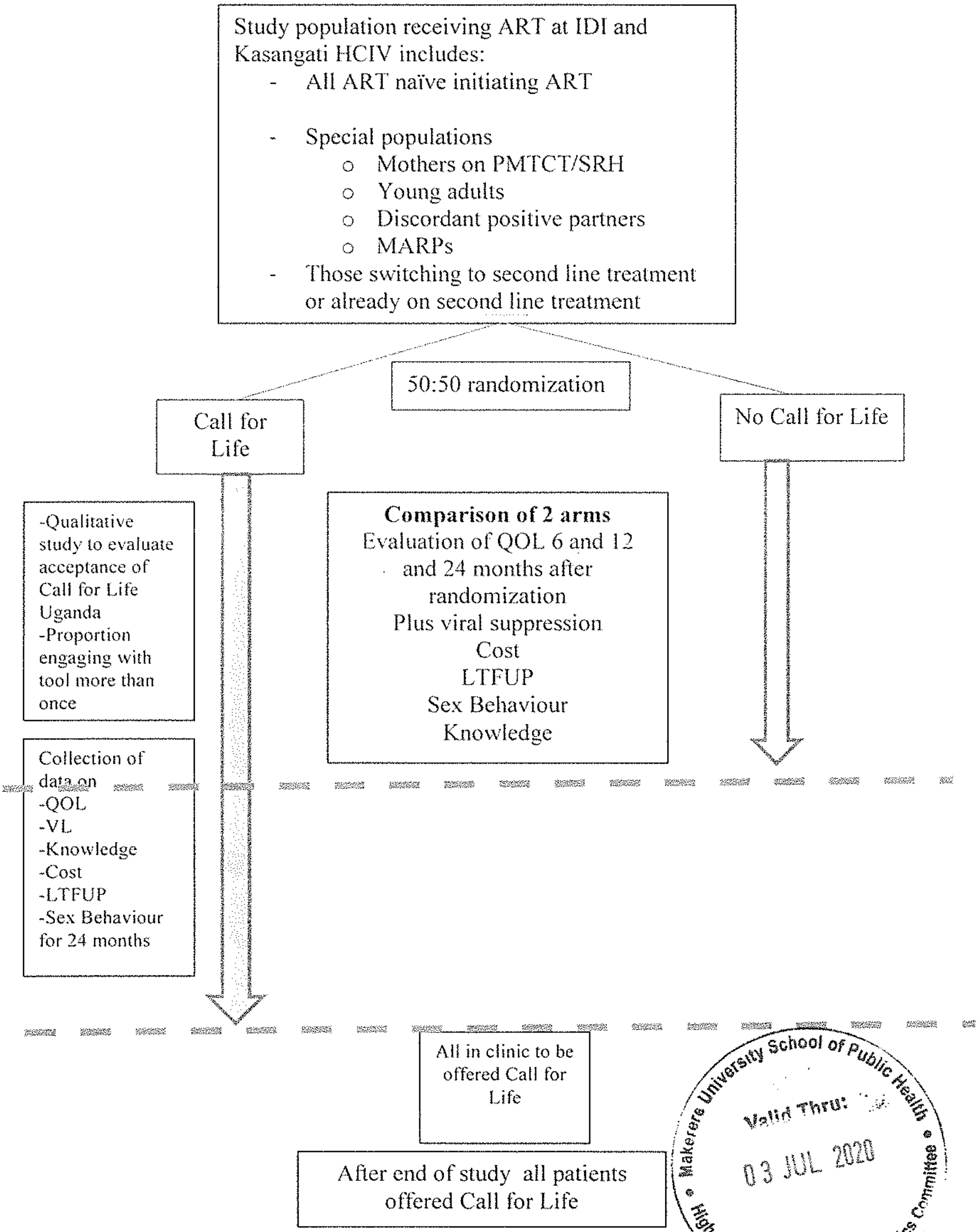

Makerere University School of Public Health • Higher Degrees, Research and Ethics Committee •
Valid Thru:
03 JUL 2020

7. ASSESSMENTS

7.1. Safety

Safety monitoring for this study will focus on unanticipated problems involving risks to participants, including unanticipated problems that meet the definition of a serious adverse event.

7.1.1. Unanticipated Problems

Unanticipated problems involving risks to participants are defined as, any incident, experience, or outcome that meets **all** of the following criteria:

- Unexpected in terms of nature, severity, or frequency given (a) the research procedures that are described in the protocol-related documents, such as the IRB-approved research protocol and informed consent document; and (b) the characteristics of the participant population being studied;
- Related or possibly related to participation in the research (possibly related means there is a reasonable possibility that the incident, experience, or outcome may have been caused by a procedures involved in the research); and
- Suggests that the research places participants or others at a greater risk of harm (including physical, psychological, economic, or social harm) than was previously known or recognized.

7.2. Pregnancy Testing

Pregnancy testing will be performed if required as standard of care at IDI, i.e. at the woman's request or missed menstrual period. Pregnant women will not be withdrawn from the study.

7.3. Post-Recruitment Illness

Patients with post-recruitment illness will be managed in accordance with standard of care in Uganda's public health sector.

Makerere University School of Public Health • Higher Degrees, Research and Ethics Committee •
Valid Thru:
03 JUL 2020

8. ADVERSE EVENT REPORTING

8.1. Adverse Events

During each study visit the study clinician will assess AEs which may have occurred since the previous visit. ART toxicities and opportunistic infections will be entered in the electronic medical records as per standard of care, as well as other outcomes (e.g: death, hospitalization).

The investigators will generate and submit annual reports summarizing these adverse events.

8.2. Serious Adverse Events

An SAE is any untoward medical occurrence at any dose that:

Results in death; Is life-threatening (immediate risk of death); Requires inpatient hospitalization or prolongation of existing hospitalization; Results in persistent or significant disability/incapacity (substantial disruption of the ability to conduct normal life functions); Results in congenital anomaly/birth defect. All SAEs will be reported to the IRB within 7 working days from the day the site gets awareness of the SAE.

8.3. Severity Assessment

This is not an intervention study and no specific drug will be investigated. Patients will be enrolled in the study to document in details the outcomes occurring during their care at the IDI clinic.

8.4. Causality Assessment

Since we are not testing any specific intervention/drug/device causality cannot be assessed

8.5. Reporting Requirements

The investigators will generate and submit annual reports summarizing AEs.

Since the study does not test any investigational drug or intervention, and causality assessment is not applicable, SAEs will also be reported to the SRC, IRB and the Uganda National Council of Science and Technology (UNCST) within 7 working days of site awareness.

9. DATA ANALYSIS/STATISTICAL METHODS

9.1. Sample Size Determination

Based on a pre and post ART assessment in Burkino Faso and Uganda we anticipate that the quality of life score (MOS-HIV) in ART naïve patients will increase at least by 15 points for physical health summary (PHS) between baseline and 12 months ART use (34, 35). We estimate there will be an additional 5 points improvement in those receiving C4L™ which would give 20-point improvement for those on C4L™ and 15 points for those not on call for Life. In those already on ART we estimate an improvement for those established on ART with a 5-point improvement for those on C4L™ and nil for those not accessing C4L™. We also estimate a 5-point increase in mental health score in those receiving C4L™ compared to no change in those without

Makerere University School of Public Health • Higher Degrees, Research and Ethics Committee
03 JUL 2020

C4L™ starting ART, and a lower improvement of 2 points in mental health score in those already on ART. The study is powered on the PHS for QOL. For a power of 0.9 and precision of 0.05 we would need a minimum of 273 in each arm (overall 546) patients, if the mean PHS among patients with C4L was 58.2 compared to 55.2 among patients without C4L. We will include a 10% LTFUP/mortality rate to give a sample size of 600.

The composition of the study participants is estimated as 300 patients at Kasangati and 300 patients at IDI.

With the proposed sample size, the numbers above depict over 90% power to test even the smallest differences in mean PHS at 12 months in the group of patients with and without C4L. We also anticipate that we will be able to detect changes in MHS QOL with this sample size.

9.2. Analysis of Endpoints

9.2.1. Analysis of Primary Endpoint

Scales to measure quality of life

There are numerous measures of quality of life. For this study we have chosen to use the Medical Outcomes Study (MOS-HIV) Health Survey which is the most widely used health related HRQoL measure in PLHIV(36).

A recent review of the HRQoL measures currently in use in HIV/AIDS clinical trials found the MOS-HIV to be one of the two most suitable HIV targeted HRQoL measures [2]. The other measure was the Functional Assessment of HIV Infection (FAHI)(37). The MOS-HIV scale was chosen because it has been validated in various settings including Uganda, has a Luganda language version, and was found to be useful in assessing HRQoL in PLHIV(38-40). In an HIV population in Uganda the MOS-HIV was reported to have Crohnbach's α coefficients > 0.70 for five out of the eight multi-item scale and factor analysis supported the underlying physical and mental health dimensions (38). In addition, the measure can be self-administered in ≤10 minutes.

The MOS-HIV measures HRQoL in 11 areas: health perceptions, bodily pain, physical function, role function, social function, mental function, vitality, health distress, cognitive function, QoL, and health transition. One scores scale in a range from 0–100 with a higher score implying better health (41). In addition to these subscales, a Physical Health Summary score (PHS) and Mental Health Summary score (MHS) can be calculated.

Intra patient in QoL scores will be compared using Paired T-tests and overall change will be investigated. The effect of calendar time on QoL life scores will be accounted for during analysis. Furthermore, the changes and or effect of the intervention on different sub-groups of HIV positives patients will be carried, for example subgroups group analysis by type and duration of ART exposure time from ART initiation, gender differences and other categories. All analysis will use robust standard errors to account for individual patient level variances/correlations, as well as cluster

Makerere University School of Public Health
03 JUL 2020
Higher Degrees, Research and Ethics Committee

(facility) correlation. The effect of the intervention will also be compared between IDI and Kasangati sites. All analysis will be conducted using STATA, USA.Analysis of Secondary Endpoints.

Appendix 18 has a summary of the QOL statistical analysis as per the DSMB terms of reference

Analysis of secondary endpoints

1) We will undertake analysis of MHS as discussed in the primary endpoint (PHS) above

2) We will undertake an intention to treat analysis of virological outcomes (logistic regression) at 6, 12, 18 and 24 months. A viral load >50 c/ml will be considered to be virological failure

3) We will undertake an intention to treat analysis of lost to follow up (Cox regression). Lost to follow will be defined as not attending appointment for >3 months.

4) We will compare knowledge about HIV, ART and associated health conditions which will be delivered as messages in the health tips section of Call for Life Uganda™ in both arms at 6, 12, 18 and 24 months of Call for Life Uganda™ use. We will add additional questions to the MOSHIV tool will be adapted for each key population.

5) We will assess perceptions and attitudes to the Call for Life Uganda™ tool thorough focus group discussions and Key informant interviews, targeting each special population. We will use purposeful sampling and will continue until no new themes emerge. We will use NVIVO software for coding of themes.

6) Cost analysis along with the study evaluation; appropriate and detailed cost evaluation will also be performed. It is anticipated that if the intervention is proved effective, a cost effective analysis would contribute to developing a sustainable scale up strategy for similar service across Uganda.
The overarching research questions addressed by this evaluation would be:
A) From a societal and health care perspective, is the Call for Life Uganda™ intervention, in comparison with usual care, preferable in terms of costs, effects and utilities?
B) What is the incremental cost-effectiveness ratio (ICER) of Call for Life Uganda™ in comparison with usual care?
C) In comparison with usual care, is the Call for Life Uganda™ intervention preferable in terms of cost and utilities during the remaining life expectancy of the study population? (model based economic evaluation)

7) We will undertake an intention to treat analysis of attendance (Cox regression). Failure to attend will be defined as not attending appointment within 3 working days of scheduled attendance in each arm.

Makerere University School of Public Health • Higher Degrees, Research and Ethics Committee •
03 JUL 2020

8) For the primary outcome, we will undertake sub-analysis for the following groups;
   a. Pregnant women already receiving ART or starting ART.
   b. Male and female positive partners in discordant couples already receiving ART or starting ART with Call for Life Uganda™
   c. Male and female young people (aged 15 to 24 years) already receiving ART or starting ART with Call for Life Uganda™
   d. Male and female patients already receiving second line ART or starting second line ART with Call for Life Uganda™
   e. Adult patients starting ART

We intend to report the study by using standard reporting methods for randomized trials (COHORT) but also to follow the newly published mERA guidelines for reporting mHealth tools which include the outcomes in the table below(33).

Table 1 | mHealth evidence reporting and assessment (mERA) guidelines, including mHealth essential criteria

Criteria	Item no	Notes	Page no where item is reported
Infrastructure (population level)	1	Clearly presents the availability of infrastructure to support technology operations in the study location. This refers to physical infrastructure such as electricity, access to power, connectivity etc. in the local context. Reporting X% network coverage rate in the country is insufficient if the study is not being conducted at the country level	
Technology platform	2	Describes and provides justification for the technology architecture. This includes a description of software and hardware and details of any modifications made to publicly available software	
Interoperability/ Health information systems (HIS) context	3	Describes how mHealth intervention can integrate into existing health information systems. Refers to whether the potential of technical and structural integration into existing HIS or programme has been described irrespective of whether such integration has been achieved by the existing system	
Intervention delivery	4	The delivery of the mHealth intervention is clearly described. This should include frequency of mobile communication, mode of delivery of intervention (that is, SMS, face to face, interactive voice response), timing and duration over which delivery occurred	
Intervention content	5	Details of the content of the intervention are described. Source and any modifications of the intervention content is described	
Usability/content testing	6	Describe formative research and/or content and/or usability testing with target group(s) clearly identified, as appropriate	
User feedback	7	Describes user feedback about the intervention or user satisfaction with the intervention. User feedback could include user opinions about content or user interface, their perceptions about usability, access, connectivity, etc	
Access of individual participants	8	Mentions barriers or facilitators to the adoption of the intervention among study participants. Relates to individual level structural, economic and social barriers or facilitators to access such as affordability, and other factors that may limit a user's ability to adopt the intervention	
Cost assessment	9	Presents basic costs assessment of the mHealth intervention from varying perspectives. This criterion broadly refers to the reporting of some cost considerations for the mHealth intervention in lieu of a full economic analysis. If a formal economic evaluation has been undertaken, it should be mentioned with appropriate references. Separate reporting criteria are available to guide economic reporting	
Adoption inputs/ programme entry	10	Describes how people are informed about the programme including training, if relevant. Includes description of promotional activities and/or training required to implement the mHealth solution among the user population of interest	
Limitations for delivery at scale	11	Clearly presents mHealth solution limitations for delivery at scale	
Contextual adaptability	12	Describes the adaptation, or not, of the solution to a different language, different population or context. Any tailoring or modification of the intervention that resulted from pilot testing/usability assessment is described	
Replicability	13	Detailed intervention to support replicability. Clearly presents the source code/screenshots/flowcharts of the algorithms or examples of messages to support replicability of the mHealth solution in another setting	
Data security	14	Describes the data security procedures/confidentiality protocols	
Compliance with national guidelines or regulatory statutes	15	Mechanism used to assure that content or other guidance/information provided by the intervention is in alignment with existing national/regulatory guidelines and is described	
Fidelity of the intervention	16	Was the intervention delivered as planned? Describe the strategies employed to assess the fidelity of the intervention. This may include assessment of participant engagement, use of backend data to track message delivery and other technological challenges in the delivery of the intervention	

Radomisation

Patients will randomly be assigned to either one of the two arms, that is, with and without CDL. To ensure that all patients in each of the arms are represented and equally balanced out in both arms, the study will use stratified randomization where each of the categories of patients at the start of the study will form the strata on interest. Within each stratum, the patients will be randomly assigned to C4L or no C4L. Randomization lists will be generated by an independent statistician and kept under lock and key in the two sites.

Makerere University School of Public Health
Valid Thru:
03 JUL 2020
Higher Degrees, Research and Ethics Committee

9.3. Interim Analysis

Interim analyses will be undertaken after sufficient patients have reached the the 6 month and one-year time-points to inform the study team on change in QOL and assess the impact of the intervention. This will be undertaken by a Data and Safety Monitoring board of 5 members appointed, including a clinical trials expert, statistician and a member with qualitative methods expertise. This board was appointed by 28th February 2017, and the membership composition is attached in the appendix. However, the study will continue to collect information to the 24-month time point, unless there is a negative effect of the intervention at 6 or 12 months.

DSMB Membership Composition (See Appendix 18)

10. QUALITY CONTROL AND QUALITY ASSURANCE

During study conduct periodic monitoring may be conducted to ensure that the protocol and Good Clinical Practices (GCPs) are being followed. The monitors may review source documents to confirm that the data recorded on CRFs is accurate. Additionally, the study site may be subject to review by the Institutional Review Board (IRB) and/or to inspection by appropriate regulatory authorities.

11. DATA HANDLING/RECORD RETENTION

11.1. Source documents including Case Report Forms (CRF)/Electronic Data Record

Participant research records should contain all of the following elements:
- Basic participant identifiers such as PTID or initials
- Documentation that the participant provided written informed consent to participate in the study prior to the conduct of any study procedures
- Documentation that the participant met the study's eligibility criteria
- A record of the participant's random assignment (if applicable)
- A record of the participant's exposure to investigational products (if applicable)
- A record of all contacts, and attempted contacts, with the participant including all clinic visits, off-site visits (e.g., at home or work), and all verbal and written contacts
- A record of all procedures performed by study staff during the study
- Complete source documents
- All case report forms (CRFs) and other study data collected from the onset of screening through end of participation
- Study-related information on the participant's condition before, during, and at the conclusion of study participation, including: o subjective data obtained directly from the participant (e.g., interview responses) o objective data ascertained by study staff (e.g., exam and laboratory findings) o objective data obtained from non-study sources (e.g., medical records)

Makerere University School of Public Health • Higher Degrees, Research and Ethics Committee • Valid Thru: 03 JUL 2020

In addition to the above, all protocol deviations involving participants should be documented in participants' study records, along with reasons for the deviation and attempts to prevent or correct the deviations, if applicable.

The ICH/GCP guidance defines source data and source documentation as follows: The term "source data" refers to all information in original records and certified copies of original records of clinical findings, observations, or other activities in a clinical trial necessary for the reconstruction and evaluation of the trial. Source data are contained in source documents (original records or certified copies). The term "source documents" refers to original documents, data and records (e.g., hospital records; clinical and office charts; laboratory notes; memoranda; subjects' diaries or evaluation checklists; pharmacy dispensing records; recorded data from automated instruments; copies of transcriptions certified after verification as being accurate and complete; microfiche; photographic negatives; microfilm or magnetic media; x-rays; subject files; and records kept at the pharmacy, the laboratories, and medico-technical departments involved in the trial).

Source documents are commonly referred to as the documents — paper-based or electronic — upon which source data are first recorded. Source documentation includes original documents and certified copies that include documentation pertaining to a participant while on study.
For this study participant case history records will consist of some or all of the following:

- Narrative chart notes on ICEA at IDI or paper MOH notes at Kasangati*
- Visit checklists or flow sheets
- Laboratory reports on ICEA at IDI, or on paper at Kasangati
- Medical records or clinic charts on ICEA at IDI or on paper at Kasangati
- Data Fax CRFs
- Randomization log or other documentation (when applicable)
- Call for Life registration and Call for Life call logs, reminder logs, symptom reporting logs and configuration settings and changes
- Other source documents and non-Data Fax data collection tools or questionnaires (e.g. FGD / IDIv notes and transcription of recordings)

*Chart Notes must be used to document the following:

- Procedures performed that are not recorded on other source documents
- Pertinent data about the participant that are not recorded on other source documents
- Protocol deviations that are not otherwise captured on other source documents
- All chart notes or other tools used as source documentation must document the PTID of the study participant to whom they pertain, the identity of the study staff member who entered information, and the date of the entry.

Makerere University School of Public Health • Higher Degrees, Research and Ethics Committee
VALID THRU: 03 JUL 2020

The CRFs developed for Call for Life are designed for use with the Data Fax data Management system. CRFs are transmitted and data are entered and cleaned using the Data Fax data management system. Data Fax is a data management system that integrates fax and computer technologies for processing CRFs. The study site retains the original CRF hard copy and transmits an electronic image to the Data fax team office based at IDI Mulago. All data transmissions are stored as electronic images at the Data fax team office. Electronic transmission is accomplished by a standard fax machine via phone lines or via the Internet using an Internet-ready fax machine. Study sites must transmit completed forms to the Data fax team office as soon as possible after completion (generally within 5 days after the participant's visit, with safety information such as adverse events (AEs) sent within 24-48 hours) and respond promptly to Data fax team office Quality control (QC) reports, clinical queries, and requests for clarifications and corrections. Site data management performance, including number of QC notes, the percentage of resolved QCs, and the time it takes the site to transmit the completed CRFs to the Data fax team office, is tracked by the Data fax team office on a regular basis

Processing of CRFs at the Data fax team office

Each Data Fax CRF is identified by a barcode denoting the protocol number and type of form. Pages do not need to be faxed in sequence. Data Fax processes images by separating a fax into individual pages, adjusting each page to correct for proper alignment and rotation, and identifying each page based on the barcode information in addition to key items such as PTID and visit code. Data Fax stores each image of a CRF that has been received and tracks all versions of each CRF received, along with all associated QC notes. DataFax uses Intelligent Character Recognition (ICR) to read data from checkboxes and numbers from numerical fields and enter it into the study database. The SDMC staff review each CRF at least twice, comparing the data entered by the ICR process with the actual data image and correcting any discrepancies. Data in specified text and comment fields are entered manually as appropriate. Data fields requiring clarification or correction (e.g., missing data or out-of-range values) or clinical data on CRFs needing verification or clarification (e.g., a severity grading on an adverse event log) are flagged with QC notes that are included in QC reports regularly emailed to the Clinical Research Sites (CRSs) for review. Corrections or clarifications in response to QC notes are made on the original CRF and re-faxed to the SDMC. The QC reporting schedule is determined by the size and progress of the study and is documented in the protocol reporting plan (see Section 12.5).

CRF Completion Site staff are trained to enter data on the CRF correctly, usually during protocol-specific training. Form-specific instructions are, in most cases, printed on the back of each CRF. CRFs have been designed using standards and conventions developed by DataFax and study team. The standard elements include: participant ID format, page numbers, visit codes, and staff initial/date fields.

CRF Revisions The need for revisions to study-specific CRFs during the conduct of a study is identified by the study team. The PI and study coordinator are responsible for revising and reissuing CRFs. Revised CRF pages are given a revision date and code. If IRB/EC approval is required for new or revised CRFs, the PI and study

Makerere University School of Public Health • Higher Degrees, Research and Ethics Committee • Valid thru: 03 JUL 2020

coordinator are responsible for seeking the approval and communicating it to team members. Once approval has been obtained, the site staff are further responsible for removing and destroying all previous versions and implementing new versions according to instructions provided by the SDMC.

Storing Data Fax Forms

Data Fax forms are designed for storage in a standard two- or three-ring binder with the holes punched down the left side of the form. They may also be placed in ordinary file folders. Storage of blank CRF supplies should be done in an organized fashion, enabling a site to inventory current supply at any time during the course of a protocol.

A CRF is required and should be completed for each included subject at enrolment and during study visits. The investigator has ultimate responsibility for the collection and reporting of all clinical, safety and laboratory data entered on the CRFs and any other data collection forms (source documents) and ensuring that they are accurate, authentic / original, attributable, complete, consistent, legible, timely (contemporaneous), enduring and available when required. The CRFs must be signed by the investigator or by an authorized staff member to attest that the data contained on the CRFs is true. Any corrections to entries made in the CRFs, source documents must be dated, initialed and explained (if necessary) and should not obscure the original entry.

11.2. Record Retention

To enable evaluations and/or audits, the investigator agrees to keep records, including the identity of all participating patients (sufficient information to link records, for example CRFs and hospital records), all original signed informed consent documents, copies of all CRFs, safety reporting forms, source documents, and detailed records of treatment disposition, and adequate documentation of relevant correspondence (for example letters, meeting minutes, telephone calls reports).

Investigator records must be kept for as long as required by applicable local regulations (UNCST generally requires a minimum of 5 years). When more than one requirement can be applied, records must be maintained for the longest period provided.

11.3. Confidentiality

Clinical data will be entered into a study specific database by designated staff on a regular basis from completed Case Record Forms (CRF). Case Record Forms and other source documents will be kept in locked cabinets. Data will be entered on a regular basis to ensure that it is up to date. The database will be [illegible] on a regular basis on a secure PC, as will the pharmacokinetic database that will be received by from the laboratories. Access to database will be given to authorized personnel only (members of the immediate study team) and a log of authorized personnel will be stored in the trial master file. CRF and trial documents will be kept in locked cabinets. No

Makerere University School of Public Health Higher Degrees, Research and Ethics Committee
03 JUL 2018

participant identifying information will be disclosed in any publication or at any conference activities arising from the study.

12. ETHICS

12.1. Institutional Review Board (IRB)

It is the responsibility of the investigator to have prospective approval of the study protocol, protocol amendments, informed consent documents, and other relevant documents from the IRB. All correspondence with the IRB should be retained in the regulatory or trial master file. Copies of IRB approvals should be filed with other study documents.

12.2. Ethical Conduct of the Study

The study will be conducted in accordance with legal and regulatory requirements, as well as the general principles set forth in the International Ethical Guidelines for Biomedical Research Involving Human Subjects and the Declaration of Helsinki.

In addition, the study will be conducted in accordance with the protocol, GCP guidelines, and applicable local regulatory requirements and laws.

12.3. Subject Information and Consent

All parties will ensure protection of subject personal data and will not include subject names on any forms, reports, publications, or in any other disclosures, except where required by laws.

The informed consent document used in this study, and any changes made during the course of the study, must be prospectively approved by the IRB.

The investigator, or a person designated by the investigator, will obtain written informed consent from each subject or the subject's legal representative before any study-specific activity is performed. The investigator will retain the original of each subject's signed consent document.

12.4. Data Privacy

Any and all data generated in IDI's use of Call for Life Uganda™ shall be owned solely by IDI. Janssen and Grameen shall not be able to directly access or utilize patient data and information. Therefore, IDI will take all reasonable steps to restrict disclosure of personal data including patient-identifiable to third parties.

In addition, the following Security Measures are in place to protect Patient Privacy when using Call for Life Uganda™

- Call for Life Uganda™ database hosted locally at IDI Kampala
- Call for Life Uganda™ Web access is password protected

Makerere University School of Public Health • Higher Degrees, Research and Ethics Committee •
Valid Thru:
03 JUL 2020

- Inactivity of 30 minutes on Call for Life Uganda™ web interface will log the web user out. The user will have to re-login into Call for Life Uganda™
- Study sponsor has no access to patient level data
- Data at Call for Life Uganda™ backend is maintained in encrypted format
- History of Changes made by investigator via web interface are tracked
- History of all web access by investigator and Admin - login/logout events is maintained
- Patients calling on Call for Life Uganda™ IVR are authenticated by their registered mobile number and the PIN they enter
- Call for Life Uganda™ will initiate calls to patients only on their registered mobile numbers and authorize Call for Life Uganda™ usage only when the called person will authenticate himself with his personal PIN
- Call for Life Uganda™ does not ask the person calling its own identity till the person has been authenticated himself as a registered patient (only Call for Life Uganda™ signature music is played

13. DEFINITION OF END OF TRIAL

The trial will be considered ended at the time of the "Database Lock"

14. PUBLICATION OF STUDY RESULTS

We aim to publish in high-impact, peer-reviewed journals with focus on open-access. Full anonymity of subject's details will be maintained throughout.
We will present data at national, regional, and international congresses (poster or oral presentation) e.g. CROI (Conference on Retroviruses and Opportunistic Infections) or IAS conferences (International AIDS Society).
The study results will also be presented to officials in charge of the antiretroviral treatment program at the Ministry of Health. Study summaries will be communicated to health care workers in Uganda through the AIDS Treatment Information Centre newsletter which is published by the IDI and distributed to all health workers in the public and not-for profit sectors in Uganda.
The study will be registered on Clinicaltrials.gov.

15. CAPACITY BUILDING

We will be employing one clinical trial co-ordinator who will be trained in clinical trials management as a result of the study. This study will also build capacity in social science by Dr Rachel King who is a senior social scientist mentoring our junior research staff. Janssen will assist our statisticians at IDI with most analysis and so build capacity in this regard.

Makerere University School of Public Health • Higher Degrees Research and Ethics Committee •
Valid Thru:
03 JUL 2020

16. FUNDING

Funding has been foreseen by Janssen Global Public Health which would be made available by the Johnson & Johnson Corporate Citizenship Trust through a grant to the Uganda Academy for Health Innovation and Impact .

Makerere University School of Public Health • Higher Degrees, Research and Ethics Committee •
Valid Thru:
03 JUL 2020

Project Timeline

	Jan – June 2016	July-sept 2016	Oct-Dec 2016	Jan-March 2017	April-June 2017	July-Sept 2017	Oct-Dec 2017	Jan-March 2018	April-June 2018	July-Sept 2018	Oct-Dec 2018	Jan-March 2019	April-June 2019	Jul-Sep 2019	Oct-Dec 2019
IRB approvals / protocol training/															
Collection of baseline data - questionnaires															
Launch of Call for Life Uganda															
Enrolment of Call for Life patients IDI															
Follow up of Call for Life patients IDI															
Enrolment of Call for Life patients Kasangati															
Follow up of Call for Life patients Kasangati															
Interim data analysis/DSMB															
Final data analysis primary outcome															
Observational data collection Call for Life patients IDI															
Observational data collection Call for Life patients Kasangati															
Analysis of observational 24 month data															
Exit Interview/Assessment															

Makerere University School of Public Health • Higher Degrees, Research and Ethics Committee
Valid Thru:
03 JUL 2020

17. REFERENCES

1. UNAIDS. GLOBAL REPORT. UNAIDS report on the global AIDS epidemic 2013. http://wwwunaidsorg/en/media/unaids/contentassets/documents/epidemiology/2013/gr2013/UNAIDS_Global_Report_2013_enpdf. 2013.
2. WHO. Consolidated guidelines on general HIV care and the use of antiretroviral drugs for treating and preventing HIV infection: recommendations for a public health approach. http://appswhoint/iris/bitstream/10665/85321/1/9789241505727_engpdf. 2013:Accessed 26.09.2013.
3. Musinguzi J. Update on the State of HIV/AIDS Epidemic in Uganda - Ugandan Ministry of Health AIDS Control Programme. 5th Annual HIV Update Meeting. 2013:25.09.2013.
4. De Cock KM, El-Sadr WM. When to start ART in Africa--an urgent research priority. The New England journal of medicine. 2013;368(10):886-9.
5. WHO. Consolidated guidelines on HIV prevention, diagnosis, treatment and care for key populations. http://wwwwhoint/hiv/pub/guidelines/keypopulations/en/. 2014;Accessed 6.1.16.
6. Mannheimer SB, Matts J, Telzak E, Chesney M, Child C, Wu AW, et al. Quality of life in HIV-infected individuals receiving antiretroviral therapy is related to adherence. AIDS care. 2005;17(1):10-22.
7. Jones D, Cook R, Rodriguez A, Waldrop-Valverde D. Personal HIV knowledge, appointment adherence and HIV outcomes. AIDS and behavior. 2013;17(1):242-9.
8. Swindells S, Mohr J, Justis JC, Berman S, Squier C, Wagener MM, et al. Quality of life in patients with human immunodeficiency virus infection: impact of social support, coping style and hopelessness. International journal of STD & AIDS. 1999;10(6):383-91.
9. Douaihy A, Singh N. Factors affecting quality of life in patients with HIV infection. The AIDS reader. 2001;11(9):450-4, 60-1, 75.
10. Mugglin C, Estill J, Wandeler G, Bender N, Egger M, Gsponer T, et al. Loss to programme between HIV diagnosis and initiation of antiretroviral therapy in sub-Saharan Africa: systematic review and meta-analysis. Tropical medicine & international health : TM & IH. 2012.
11. Geng EH, Bwana MB, Muyindike W, Glidden DV, Bangsberg DR, Neilands TB, et al. Failure to initiate antiretroviral therapy, loss to follow-up and mortality among HIV-infected patients during the pre-ART period in Uganda. Journal of acquired immune deficiency syndromes. 2013;63(2):e64-71.
12. Barth RE, van der Loeff MF, Schuurman R, Hoepelman AI, Wensing AM. Virological follow-up of adult patients in antiretroviral treatment programmes in sub-Saharan Africa: a systematic review. The Lancet Infectious diseases. 2010;10(3):155-66.
13. Reda AA, Biadgilign S. Determinants of Adherence to Antiretroviral Therapy among HIV-Infected Patients in Africa. AIDS research and treatment. 2012;2012:574656.

Makerere University School of Public Health • Higher Degrees, Research and Ethics Committee •
Valid Thru:
03 JUL 2020

14. Anglaret X, Scott CA, Walensky RP, Ouattara E, Losina E, Moh R, et al. Could early antiretroviral therapy entail more risks than benefits in sub-Saharan African HIV-infected adults? A model-based analysis. Antiviral therapy. 2013;18(1):45-55.
15. Stephen Ian Walimbwa JS, Patience Nyakato, Florence Namimbi, Rosalind Parkes-Ratanshi. ACCEPTANCE OF HAART AT HIGHER CD4 IN DISCORDANT COUPLES IN UGANDA
ICASA Conference, South Africa. 2013:Submitted.
16. Gourlay A, Birdthistle I, Mburu G, Iorpenda K, Wringe A. Barriers and facilitating factors to the uptake of antiretroviral drugs for prevention of mother-to-child transmission of HIV in sub-Saharan Africa: a systematic review. Journal of the International AIDS Society. 2013;16(1):18588.
17. Ngarina M, Popenoe R, Kilewo C, Biberfeld G, Ekstrom AM. Reasons for poor adherence to antiretroviral therapy postnatally in HIV-1 infected women treated for their own health: experiences from the Mitra Plus study in Tanzania. BMC public health. 2013;13(1):450.
18. Mbuagbaw L, Thabane L, Ongolo-Zogo P, Lester RT, Mills EJ, Smieja M, et al. The Cameroon Mobile Phone SMS (CAMPS) trial: a randomized trial of text messaging versus usual care for adherence to antiretroviral therapy. PloS one. 2012;7(12):e46909.
19. Lester RT, Ritvo P, Mills EJ, Kariri A, Karanja S, Chung MH, et al. Effects of a mobile phone short message service on antiretroviral treatment adherence in Kenya (WelTel Kenya1): a randomised trial. Lancet. 2010;376(9755):1838-45.
20. Pop-Eleches C, Thirumurthy H, Habyarimana JP, Zivin JG, Goldstein MP, de Walque D, et al. Mobile phone technologies improve adherence to antiretroviral treatment in a resource-limited setting: a randomized controlled trial of text message reminders. Aids. 2011;25(6):825-34.
21. J.M.Ikeda. SMS messaging improves treatment outcome among the HIV-positive Mayan population in rural Guatemala. 19th International AIDS Conference. 2012:Abstract no. TUPE673.
22. Rodrigues R, Shet A, Antony J, Sidney K, Arumugam K, Krishnamurthy S, et al. Supporting adherence to antiretroviral therapy with mobile phone reminders: results from a cohort in South India. PloS one. 2012;7(8):e40723.
23. al dCTe. Results of a randomized controlled trial to assess the effects of a mobile SMS-based intervention on
treatment adherence in HIV/AIDS-infected Brazilian women and impressions and satisfaction with respect to incoming
messages. International journal of medical informatics.81:257–69.
24. al. AAe. Timed short messaging service improves adherence and virological outcomes in HIV-1-infected patients
with suboptimal adherence to antiretroviral therapy. . Journal of Acquired Immune Deficiency Syndromes,. 2011; 58:e113–e5.
25. Sidney K, Antony J, Rodrigues R, Arumugam K, Krishnamurthy S, D'Souza G, et al. Supporting patient adherence to antiretrovirals using mobile phone reminders: patient responses from South India. AIDS care. 2012;24(5):612-7.

Makerere University School of Public Health • Higher Degrees, Research and Ethics Committee •
Valid Thru:
03 JUL 2020

26. Mbuagbaw L, Thabane L, Ongolo-Zogo P. Opening communication channels with people living with HIV using mobile phone text messaging: Insights from the CAMPS trial. BMC research notes. 2013;6(1):131.
27. van der Kop ML, Ojakaa DI, Patel A, Thabane L, Kinagwi K, Ekstrom AM, et al. The effect of weekly short message service communication on patient retention in care in the first year after HIV diagnosis: study protocol for a randomised controlled trial (WelTel Retain). BMJ open. 2013;3(6).
28. Dean AL, Makin JD, Kydd AS, Biriotti M, Forsyth BW. A pilot study using interactive SMS support groups to prevent mother-to-child HIV transmission in South Africa. Journal of telemedicine and telecare. 2012;18(7):399-403.
29. Miller LG, Liu H, Hays RD, Golin CE, Ye Z, Beck CK, et al. Knowledge of antiretroviral regimen dosing and adherence: a longitudinal study. Clinical infectious diseases : an official publication of the Infectious Diseases Society of America. 2003;36(4):514-8.
30. Stangl AL, Bunnell R, Wamai N, Masaba H, Mermin J. Measuring quality of life in rural Uganda: reliability and validity of summary scores from the medical outcomes study HIV health survey (MOS-HIV). Quality of life research : an international journal of quality of life aspects of treatment, care and rehabilitation. 2012;21(9):1655-63.
31. Miners A, Phillips A, Kreif N, Rodger A, Speakman A, Fisher M, et al. Health-related quality-of-life of people with HIV in the era of combination antiretroviral treatment: a cross-sectional comparison with the general population. The lancet HIV. 2014;1(1):e32-40.
32. WHO. Consolidated guidelines on general HIV care and the use of antiretroviral drugs for treating and preventing HIV infection: recommendations for a public health approach. 2013.
33. Agarwal S, LeFevre AE, Lee J, L'Engle K, Mehl G, Sinha C, et al. Guidelines for reporting of health interventions using mobile phones: mobile health (mHealth) evidence reporting and assessment (mERA) checklist. BMJ. 2016;352:i1174.
34. Jaquet AG, F. Balestre, E. Ekouevi, D. Azani, JC. Bognounou, R., Dah EK, E. Dabis, F. Drabo, J. Antiretroviral treatment and quality of life in Africans living with HIV: 12-month follow-up in Burkina Faso. Journal of the International AIDS Society. 2013;6:=(18867).
35. Stangl AL, Wamai N, Mermin J, Awor AC, Bunnell RE. Trends and predictors of quality of life among HIV-infected adults taking highly active antiretroviral therapy in rural Uganda. AIDS care. 2007;19(5):626-36.
36. Tarlov AR WJJ, Greenfield S, Nelson EC, Perrin E, Zubkoff M. . The medical outcomes study: An application of methods for monitoring the results of medical care. JAMA 1989;262::925–30.
37. Clayson D WD, Quarterman P, Duprat-Lomon I, Kubin M, Coons S. A Comparative Review of Health-Related Quality-of-Life Measures for Use in HIV/AIDS Clinical Trials. Pharmacoeconomics. . 24. 2006:751–65.
38. Wu AW RD, Jacobson D, Malitz FE. Evidence for Reliability, Validity and Usefulness of the Medical Outcomes Study HIV Health Survey (MOS-HIV). . Quality of Life Research. 1997; 6:481–93.

Makerere University School of Public Health • Higher Degrees, Research and Ethics Committee •
Valid Thru:
03 JUL 2020

39. Mast T KG, Wabwire-mangen F, Black R, Sewankambo N, Serwadda D, . Measuring quality of life among HIV-infected women using a culturally adapted questionnaire in Rakai district, Uganda. AIDS care. 2004;16:81–94.

40. Epino HM RM, Kaigamba F, Hakizamungu M, Socci AR, Bagiruwigize E,. Reliability and construct validity of three health-related self-report scales in HIV-positive adults in rural Rwanda. AIDS care. 2012;24:1576–83.

41. Revicki DA SS, Wu AW. Reliability and Validity of Physical and Mental Health Summary Scores from the Medical Outcomes Study HIV Health Survey. Medical Care. 1998;36:126–37.

30. Gurol-Urganci I, de Jongh T, Vodopivec-Jamsek V, Atun R, Car J
Mobile phone messaging reminders for attendance at healthcare appointments (Review) *The Cochrane Library* 2013, Issue 12

31. Joshi et al. 2014. Supporting Treatment of People Living with HIV / AIDS in Resource Limited Settings with IVRs. Proceedings of the SIGCHI Conference on Human Factors in Computing Systems, 2014; pages 1595-1604.

Makerere University School of Public Health • Higher Degrees, Research and Ethics Committee •
Valid Thru:
03 JUL 2020

18. APPENDIX 1: DATA SAFETY AND MONITORING BOARD MEMBERS

1. Rujumba Joseph email:jrujumba@yahoo.com (Social Scientist, Makerere University College of Health Sciences, School of Medicine, Pediatrics' Dep't)
2. Sylvia Kiwuwa Muyingo email:Sylvia.muyingo@mrcuganda.org (Biostatistician, MRC-UVRI)
3. Steven Reynolds email : sjr@jhmi.edu (Medical Doctor Researcher, CDC- UVRI)
4. Andrew Kambugu email: akambugu@idi.co.ug (Physician, IDI Research Office)
5. Erisa Mwaka, email: erisamwaka@gmail.com (Orthopedic Surgeon, Bioethicist, Makerere University, College of Health Sciences, School of Biomedical Sciences)

Summary of Statistical Analysis for QOL

Item scaling	3, 5 and 6-point Likert-type scale Dichotomous yes/no
Scales	There are 11 scales in the MOS-HIV questionnaire, namely; General health, Pain, Physical functioning, Role functioning, Social functioning, Energy/Fatigue/Vitality, Mental health, Health distress, Cognitive functioning, Quality of life, and Health transition.
Scoring Procedure, Interpretation and Analysis	1. Item recoding; 11 of the 35 items in the survey require recoding.<br>2. Item scores in each scale are summed to compute raw scale scores;<br>3. Raw scale scores are linearly transformed to a 0 - 100 scale (transformed scale scores) to facilitate comparisons with other MOS-HIV Health Survey data.<br>4. For multi-item scales, mean substitution is generally used for missing items if ≤ 50 % of the items are missing.<br>Analysis is carried out using a difference-in-difference method. Difference-in-differences (DID) treatment-effect is the estimation of a given outcome variable from a pooled baseline and follow-up dataset, p-value for the treatment effect, or DID estimator will be reported at 90% and 95% confidence level.<br>• Relies on the panel structure of the data (usually two periods: based line and follow up).<br>• Control for unobservable and time invariant characteristics.<br>• Control for observable characteristics if available.

Makerere University School of Public Health • Higher Degrees, Research and Ethics Committee • Valid Thru: 03 JUL 2020

Chi square and Fishers exact test	A chi-square test for independence compares two variables in a contingency table to see if they are related. In a more general sense, it tests to see whether distributions of categorical variables differ from each another.<br>• A very small chi square test statistic means that your observed data fits your expected data extremely well. In other words, there is a relationship.<br>• A very large chi square test statistic means that the data does not fit very well. In other words, there is not a relationship.<br>In cases where the number of cases in a cell were less than 5 then the fisher's exact test was used.
ANCOVA analysis	Analysis of covariance is used to test the main and interaction effects of categorical variables on a continuous dependent variable, controlling for the effects of selected other continuous variables, which co-vary with the dependent. Analysis of covariance (ANCOVA) allows to compare one variable in 2 or more groups taking into account (or to correct for) variability of other variables, called covariates. ANCOVA analysis was carried out with the baseline scores and arm as covariates to account for their effect on the follow-up respective scores.
Linear Mixed Model	A linear mixed model will be used, adjusting for time and ARM and accounting for individual and site variability for the overall.<br>By site a linear mixed model will be done adjusting for time and ARM accounting for only individual variability.

Makerere University School of Public Health • Higher Degrees, Research and Ethics Committee •
Valid Thru:
03 JUL 2020
