# Supplementary material for: An Interactive Voice Response Software to Improve the Quality of Life of People Living With HIV in Uganda: Randomized Controlled Trial
Source: JMIR Mhealth Uhealth. 2021 Feb 11;9(2):e22229. doi: 10.2196/22229 (PMC7906832; doi:10.2196/22229)
Supplement: Multimedia Appendix 6 [file mhealth_v9i2e22229_app6.docx]

**Multimedia Appendix 6:** CONSORT-EHEALTH, and mERA checklists

**Corresponding Author *:** Dathan M. Byonanebye

**Primary Affiliation (short), City, Country *:** Makerere University School of Public Health, Kampala, Uganda

**Your e-mail address *:** dbyonanebye@musph.ac.ug

**Title of your manuscript *:** An interactive voice response software to improve the quality of life of people living with HIV in Uganda: A Randomized Controlled Trial

**Name of your App/Software/Intervention *:** CFL-Call for Life Uganda.

**Evaluated Version (if any):** “V9.3.0”, Release 2016-01-01.

**Language(s) *:** English, Kiswahili, Luganda, Runyankore

URL of your Intervention Website or App: http://www.callforlifeuganda.com/motech-platform-server/module/cfl-web/resources/login.html#/

**Accessibility *:** Access to the software is not open to the public.

**Primary Medical Indication/Disease/Condition *:** HIV/AIDS

**Primary Outcomes measured in trial *:** Change in Quality of Life

**Secondary/other outcomes:** viral suppression, appointment keeping.

Recommended "Dose" *: Approximately Daily

**Approx. Percentage of Users (starters) still using the app as recommended after 3 months *:** 41-50%

**Overall, was the app/intervention effective? *:** There was a statistical difference in the primary outcome in participants with high use of the application versus those who had minimal use of the intervention.

**Article Preparation Status/Stage *:** submitted to a journal and accepted, but not published yet published.

**Journal *:** JMIR mHealth and UHealth

**Is this a full-powered effectiveness trial or a pilot/feasibility trial? *:** Fully powered.

**Manuscript tracking number *:** 22229

**TITLE AND ABSTRACT**

**1a) TITLE: Identification as a randomized trial in the title**

**1a) Does your paper address CONSORT item 1a?** *: Yes

**1a-i) Identify the mode of delivery in the title:** Yes

*“An interactive voice response software to improve the quality of life of people living with HIV in Uganda: A Randomized Controlled Trial”.*

**1a-ii) Non-web-based components or important co-interventions in title**

**Does your paper address subitem 1a-ii?** Not applicable for this paper

**1a-iii) Primary condition or target group in the title:** "People living with HIV/AIDS (PLWH)"

**1b) ABSTRACT: Structured summary of trial design, methods, results, and conclusions**

**1b-i) Key features/functionalities/components of the intervention and comparator in the METHODS section of the ABSTRACT**

**Does your paper address subitem 1b-i? *:** Yes “, “*Within the Call for Life (CFL) study, ART-experienced and ART naïve PLHIV commencing ART were randomized (1:1 ratio) to control (no IVR support) or intervention arm (daily adherence and pre-appointment reminders, health information tips and option to report symptoms)"* and also “*The primary and secondary outcomes were to determine the impact of interactive voice response (IVR) technology on Medical Outcomes Study HIV QoL scores and viral suppression at 12 months, respectively*”

**1b-ii) Level of human involvement in the METHODS section of the ABSTRACT**

**Does your paper address subitem 1b-ii?** Yes, "*delivered via automated intervention interactive voice response or short message service*”.

**1b-iii) Open vs. closed, web-based (self-assessment) vs. face-to-face assessments in the METHODS section of the ABSTRACT.**

**Does your paper address subitem 1b-iii?** Yes

**1b-iv) RESULTS section in abstract must contain use data**

**Does your paper address subitem 1b-iv?** Yes, “*Overall, 600 participants (413 female, 68.8%) were enrolled and followed-up for 12 months. In the intervention arm of 300 participants, 298 (99.3%) opted for IVR and 2 (0.7%) chose SMS text messaging as the mode of receiving reminders and health tips. At 12 months, there was no overall difference in the QoL between the intervention and control arms (DID=0.0; P=.99) or HIV RNA (DID=0.01; P=.94). At 12 months, 124 of the 256 (48.4%) active participants had picked up at least 50% of the calls. In the active intervention participants, high users (received >75% of reminders) had overall higher QoL compared to low users (received <25% of reminders) (92.2 versus 87.8, P=.02). Similarly, high users also had higher QoL scores in the mental health domain (93.1 versus 86.8, P=.008) and better appointment keeping. Similarly, participants with moderate use (51%-75%) had better viral suppression at 12 months (80/94, 85% versus 11/19, 58%, P=.006).”*

1b-v) CONCLUSIONS/DISCUSSION in abstract for negative trials

Conclusions/Discussions in abstract for negative trials: Discuss the primary outcome - if the trial is negative (primary outcome not changed), and the intervention was not used, discuss whether negative results are attributable to lack of uptake and discuss reasons.

**Does your paper address subitem 1b-v?** Yes, *“Overall, there was high uptake and acceptability of the IVR tool. While we found no overall difference in the QoL and viral suppression between study arms, people living with HIV with higher usage of the tool showed greater improvements in QoL, viral suppression, and appointment keeping. With the declining resources available to HIV programs and the increasing number of people living with HIV accessing ART, IVR technology could be used to support patient care. The tool may be helpful in situations where physical consultations are infeasible, including the current COVID epidemic.”*

**INTRODUCTION**

**2a) In INTRODUCTION: Scientific background and explanation of the rationale**

**2a-i) Problem and the type of system/solution**

**Does your paper address subitem 2a-i? *** Yes, "However, provision of continuous support to ensure sustained adherence is challenging and requires intensive health care worker time and resources. Therefore, there is need for patient-centered adherence systems that do not add additional burden to the already constrained health systems, especially in the low- and middle-income countries with a high burden of HIV."

2a-ii) Scientific background, rationale: What is known about the (type of) system.

**Does your paper address subitem 2a-ii? ***

Copy and paste relevant sections from the manuscript (include quotes in quotation marks "like this" to indicate direct quotes from your manuscript), or elaborate on this item by providing additional information, not in the ms, or briefly explain why the item is not applicable/relevant for your study.

"*Partly because of the increasing access to mobile technology in the most burdened countries,[7] mobile health technologies are increasingly leveraged to support PLHIV. However, there is limited evidence on the impact of mHealth tools on QoL in PLHIV, especially in LMICs. The majority of mHealth tools evaluated in LMICs have used short message service (SMS) interventions with mixed results.[8] However, adoption and impact of SMS-interventions in countries with low literacy rates is likely to be low.* "

**2b) In INTRODUCTION: Specific objectives or hypotheses**

Does your paper address CONSORT subitem 2b? *Yes " *We hypothesized that IVR-based technology could provide motivational information and reminders to people living with HIV and ultimately improve ART adherence, QoL, and viral suppression.* "

**METHODS**

**3a) Description of trial design (such as parallel, factorial) including allocation ratio**

**Does your paper address CONSORT subitem 3a?** * Yes, "*Eligible patients were randomized to either control (standard of care) or intervention arms (1:1 ratio) in this open label study.* "

**3b) Important changes to methods after trial commencement (such as eligibility criteria), with reasons**

**Does your paper address CONSORT subitem 3b? ***Yes, "*The study was halted between January 20 and February 2, 2018, due to a software failure. There were no confidentiality breaches during this software failure, and a protocol deviation was reported to the institutional review board. Upon fixing the technical failure, participants were interviewed on whether they wanted to re-enrol*”.

**3b-i) Bug fixes, Downtimes, Content Changes**

Does your paper address subitem 3b-i? Yes, " *The major challenges experienced during study implementation included internet and system failures and the need for security upgrades (Multimedia Appendix 1). As a result, the system was iteratively upgraded from version 1.0.0 to version 9.3.0, before and during use in the study. The changes aimed at optimizing usability and security, not the intervention delivered, as summarized in Multimedia Appendix 2.* "

**4a) Eligibility criteria for participants**

**Does your paper address CONSORT subitem 4a? * Yes,** " *ART-naïve and ART-experienced people living with HIV were consecutively screened, and participants were eligible for enrollment if they belonged to any of the following categories: ART-naïve adults or ART-experienced people living with HIV, including key populations (sex workers and men who have sex with men), young adults (18-24 years), pregnant and breastfeeding mothers, and people living with HIV in discordant relationships*. "

**4a-i) Computer / Internet literacy**

**Computer / Internet literacy is often an implicit “de facto” eligibility criterion - this should be explicitly clarified.**

**Does your paper address subitem 4a-i?** Yes,” " *Participants did not require smartphones. They received and made calls using any mobile phones, including feature phones*."

4a-ii) Open vs. closed, web-based vs. face-to-face assessments:

Open vs. closed, web-based vs. face-to-face assessments: Mention how participants were recruited (online vs. offline), e.g., from an open access website or from a clinic, and clarify if this was a purely web-based trial, or there were face-to-face components (as part of the intervention or for assessment), i.e., to what degree got the study team to know the participant. In online-only trials, clarify if participants were quasi-anonymous and whether having multiple identities was possible or whether technical or logistical measures (e.g., cookies, email confirmation, phone calls) were used to detect/prevent these.

Does your paper address subitem 4a-ii? * Yes," *Participants were physically evaluated at baseline and months 6 and 12. At each time point, the study team collected data on sociodemographics (age, sex, marital status) and treatment history (ART status, duration on ART, ART regimen, and HIV RNA*"

**4a-iii) Information giving during the recruitment**

Information given during recruitment. Specify how participants were briefed for recruitment and in the informed consent procedures (e.g., publish the informed consent documentation as appendix, see also item X26), as this information may have an effect on user self-selection, user expectation and may also bias results.

**Does your paper address subitem 4a-iii?** Yes, " *At baseline, participants in the intervention arm were trained on how to initiate and receive calls. Participants were asked at each appointment if they were experiencing challenges with the IVR calls. Call completion rates were reviewed weekly.* "

4b) Settings and locations where the data were collected

Does your paper address CONSORT subitem 4b? * Yes, "*The study was conducted at two HIV clinics. The Infectious Diseases Institute (IDI) clinic is a specialist urban HIV clinic located within the National Mulago Hospital complex and serves more than 8000 people living with HIV. Kasangati Health Centre IV is a government-owned clinic in peri-urban Kampala and serves approximately 5000 people living with HIV. At both clinics, comprehensive HIV care and treatment services were provided according to the WHO and national guidelines for HIV treatment [14,15]. Nurse counselors physically provide face-to-face patient adherence support during clinic visits with no option for remote support*. "

4b-i) Report if outcomes were (self-)assessed through online questionnaires

Clearly report if outcomes were (self-)assessed through online questionnaires (as common in web-based trials) or otherwise.

**Does your paper address subitem 4b-i? *,** Yes "*Participant evaluation was physically conducted at baseline, months 6, 12, 18 and 24*."

**4b-ii) Report how institutional affiliations are displayed**

Report how institutional affiliations are displayed to potential participants [on ehealth media], as affiliations with prestigious hospitals or universities may affect volunteer rates, use, and reactions with regards to an intervention. (Not a required item – describe only if this may bias results)

**Does your paper address subitem 4b-ii?** Yes, This is shown in the consent.

**5) The interventions for each group with sufficient details to allow replication, including how and when they were actually administered**

**5-i) Mention names, credential, affiliations of the developers, sponsors, and owners**

Mention names, credential, affiliations of the developers, sponsors, and owners [6] (if authors/evaluators are owners or developer of the software, this needs to be declared in a “Conflict of interest” section or mentioned elsewhere in the manuscript).

**Does your paper address subitem 5-i?** Yes, *“The technology evaluated in this study was CFL, a software that is based on open-source Mobile Technology for Community Health (MoTeCH). MoTeCH was initially developed by the Grameen Foundation and the University of Southern Maine with the support of Janssen, the Pharmaceutical Companies of Johnson & Johnson. Before this study, the software was used in India and Ghana [16,17]. The initial software was called Treatment Advice by Mobile Alerts (TAMA) [17]. Following the adaptation of TAMA for use by people living with HIV in Uganda, the local system was named Call for Life”.*

**5-ii) Describe the history/development process**

Does your paper address subitem 5-ii? Yes, “*The technology evaluated in this study was CFL, a software that is based on open-source Mobile Technology for Community Health (MoTeCH). MoTeCH was initially developed by the Grameen Foundation and the University of Southern Maine with the support of Janssen, the Pharmaceutical Companies of Johnson & Johnson. Before this study, the software was used in India and Ghana [16,17]. The initial software was called Treatment Advice by Mobile Alerts (TAMA) [17]. Following the adaptation of TAMA for use by people living with HIV in Uganda, the local system was named Call for Life.”*

**5-iii) Revisions and updating**

Revisions and updating. Clearly mention the date and/or version number of the application/intervention (and comparator, if applicable) evaluated, or describe whether the intervention underwent major changes during the evaluation process, or whether the development and/or content was “frozen” during the trial. Describe dynamic components such as news feeds or changing content which may have an impact on the replicability of the intervention (for unexpected events see item 3b).

**Does your paper address subitem 5-iii?** Yes, *“The major challenges experienced during study implementation included internet and system failures and the need for security upgrades (Multimedia Appendix 1). As a result, the system was iteratively upgraded from version 1.0.0 to version 9.3.0, before and during use in the study. The changes aimed at optimizing usability and security, not the intervention delivered, as summarized in Multimedia Appendix 2.”*

**5-iv) Quality assurance methods**

Provide information on quality assurance methods to ensure accuracy and quality of information provided [1], if applicable.

Does your paper address subitem 5-iv? Yes, “*All study data were double-encrypted by CFL. All clinic data of people living with HIV remained on the local IDI servers as per Uganda data protection guidelines. Communication between the CFL browser and the server was encrypted using 128-bit Secure Sockets Layer. CFL system servers were hosted by Amazon Web Services (AWS) and secured by Amazon virtual private cloud and AWS web firewalls. At the same time, data were protected from virus threats using Bitdefender antivirus technology.”*

**5-v) Ensure replicability by publishing the source code, and/or providing screenshots/screen-capture video, and/or providing flowcharts of the algorithms used**

Ensure replicability by publishing the source code, and/or providing screenshots/screen-capture video, and/or providing flowcharts of the algorithms used. Replicability (i.e., other researchers should in principle be able to replicate the study) is a hallmark of scientific reporting.

**Does your paper address subitem 5-v?** Yes, *“The software evaluated was Call for Life Uganda, an IVR technology that is based on the Mobile Technology for Community Health open-source software.”* **and** “Multimedia Appendix 4 shows the call flow diagram for the CFL software.”

**5-vi) Digital preservation**

Digital preservation: Provide the URL of the application, but as the intervention is likely to change or disappear over the course of the years; also make sure the intervention is archived (Internet Archive, webcitation.org, and/or publishing the source code or screenshots/videos alongside the article). As pages behind login screens cannot be archived, consider creating demo pages which are accessible without login.

**Does your paper address subitem 5-vi?** Yes, *The call for life software URL* [*www.callforlifeuganda.com*](http://www.callforlifeuganda.com) *is referenced in the paper.*

**5-vii) Access**

Access: Describe how participants accessed the application, in what setting/context, if they had to pay (or were paid) or not, whether they had to be a member of specific group. If known, describe how participants obtained “access to the platform and Internet” [1]. To ensure access for editors/reviewers/readers, consider to provide a “backdoor” login account or demo mode for reviewers/readers to explore the application (also important for archiving purposes, see vi).

**Does your paper address subitem 5-vii?** * Yes. ***“****The system allows automatic interaction with patients using voice and tone input via a keypad (IVR) or by SMS text messaging using simple phones (GSM-2/feature). CFL was integrated with the patient health information management systems used for HIV care in Uganda, so as to obtain appointment dates and ART regimens.”*

**5-viii) Mode of delivery, features/functionalities/components of the intervention and comparator, and the theoretical framework**

Describe mode of delivery, features/functionalities/components of the intervention and comparator, and the theoretical framework [6] used to design them (instructional strategy [1], behaviour change techniques, persuasive features, etc., see e.g., [7, 8] for terminology). This includes an in-depth description of the content (including where it is coming from and who developed it) [1],” whether [and how] it is tailored to individual circumstances and allows users to track their progress and receive feedback” [6]. This also includes a description of communication delivery channels and – if computer-mediated communication is a component – whether communication was synchronous or asynchronous [6]. It also includes information on presentation strategies [1], including page design principles, average amount of text on pages, presence of hyperlinks to other resources, etc. [1].

**Does your paper address subitem 5-viii?** * Yes, *“To increase the adoption, replication, and impact of the intervention, we used the criteria and taxonomy suggested by Tabak et al [10] and selected the information, motivation, and behavioral skills (IMB) model of behavioral change [11] as the theory for the intervention (Figure 1*)” **and**” Participants chose the preferred languages, time, and frequency of receiving reminders. For security, both outbound and inbound calls played music until the participant entered a personal identification number (PIN) unique to them. Participants chose their preferred “health topics”, from which the system randomly shuffled and randomly played different health tips for each call (Multimedia Appendix 3)"

**5-ix) Describe use parameters**

Describe use parameters (e.g., intended “doses” and optimal timing for use). Clarify what instructions or recommendations were given to the user, e.g., regarding timing, frequency, heaviness of use, if any, or was the intervention used ad libitum.

**Does your paper address subitem 5-ix? Yes, *“****Participants chose the preferred languages, time, and frequency of receiving reminders.”*

**5-x) Clarify the level of human involvement**

Clarify the level of human involvement (care providers or health professionals, also technical assistance) in the e-intervention or as co-intervention (detail number and expertise of professionals involved, if any, as well as “type of assistance offered, the timing and frequency of the support, how it is initiated, and the medium by which the assistance is delivered”. It may be necessary to distinguish between the level of human involvement required for the trial, and the level of human involvement required for a routine application outside of a RCT setting (discuss under item 21 – generalizability).

**Does your paper address subitem 5-x?** Yes, *”In addition, intervention participants had an option to call a toll-free line and report symptoms or drug side effects. Such patient-triggered calls could generate alerts that would prompt health care workers to call back within 24 hours.”* **And** *"Patients were regularly interviewed if they were experiencing challenges with the IVR calls. " Participants were asked at each appointment if they were experiencing challenges with the IVR calls. Call completion rates were reviewed weekly. Patients with a blocked PIN were contacted by the study medical team within one week to reset the PIN.”*

**5-xi) Report any prompts/reminders used**

Report any prompts/reminders used: Clarify if there were prompts (letters, emails, phone calls, SMS) to use the application, what triggered them, frequency etc. It may be necessary to distinguish between the level of prompts/reminders required for the trial, and the level of prompts/reminders for a routine application outside of a RCT setting (discuss under item 21 – generalizability).

**Does your paper address subitem 5-xi? *** *Yes, "Patients received IVR call or SMS appointment reminders on or prior to the scheduled appointment date". "On the intervention arm PLHIV received SOC plus daily adherence IVR call or SM"*

**5-xii) Describe any co-interventions (incl. training/support)**

Describe any co-interventions (incl. training/support): Clearly state any interventions that are provided in addition to the targeted eHealth intervention, as ehealth intervention may not be designed as stand-alone intervention. This includes training sessions and support [1]. It may be necessary to distinguish between the level of training required for the trial, and the level of training for a routine application outside of a RCT setting (discuss under item 21 – generalizability.

**Does your paper address subitem 5-xii?** * *Yes, "face-to-face facility appointments"*

**6a) Completely defined pre-specified primary and secondary outcome measures, including how and when they were assessed**

**Does your paper address CONSORT subitem 6a?** * Yes, " *The primary outcome was the difference in the differences in QoL between the study arms at month 12."*

**6a-i) Online questionnaires: describe if they were validated for online use and apply CHERRIES items to describe how the questionnaires were designed/deployed**

If outcomes were obtained through online questionnaires, describe if they were validated for online use and apply CHERRIES items to describe how the questionnaires were designed/deployed [9].

**Does your paper address subitem 6a-i?** Not applicable. All interviews were face-face.

**6a-ii) Describe whether and how “use” (including intensity of use/dosage) was defined/measured/monitored**

Describe whether and how “use” (including intensity of use/dosage) was defined/measured/monitored (logins, logfile analysis, etc.). Use/adoption metrics are important process outcomes that should be reported in any ehealth trial.

**Does your paper address subitem 6a-ii?** Yes, *“Additionally, we compared the outcomes within the intervention arm according to the intensity of use of the system (proportion of users receiving reminders): low users (<25% calls answered), fairly low users (26%-50% of calls answered), moderate users (51%-75% answered) and high users (>75% calls answered).”*

**6a-iii) Describe whether, how, and when qualitative feedback from participants was obtained**

Describe whether, how, and when qualitative feedback from participants was obtained (e.g., through emails, feedback forms, interviews, focus groups).

**Does your paper address subitem 6a-iii?** Yes,but this paper focuses on quantitative outcomes. Nevertheless, ”Qualitative interviews suggested that people living with HIV became attached to the “voice” and felt as if they were better cared for.”

**6b) Any changes to trial outcomes after the trial commenced, with reasons.**

**Does your paper address CONSORT subitem 6b? *** *Yes, “The major challenges experienced during study implementation included internet and system failures and the need for security upgrades (Multimedia Appendix 1). As a result, the system was iteratively upgraded from version 1.0.0 to version 9.3.0, before and during use in the study. The changes aimed at optimizing usability and security, not the intervention delivered, as summarized in Multimedia Appendix 2.”*

**7a) How sample size was determined.**

**7a-i) Describe whether and how expected attrition was taken into account when calculating the sample size**

**Does your paper address subitem 7a-i?** *Yes, “Based on pre- and post-ART assessments of QoL in people living with HIV in Burkina Faso [7] and Uganda [20], we anticipated a 15-point difference in overall QoL following ART in people living with HIV in the control and intervention arms. We also estimated that there would be an additional 5-point improvement in the QoL in those receiving CFL. Therefore, we expected an overall difference of 5 points in ART-experienced people living with HIV in the intervention versus control arms. For a power of 90% and precision of 0.05, we needed a minimum of 273 patients in each arm (overall 546) to detect a 5-point difference in the QoL in the intervention versus the control arm. The estimated sample size was adjusted for the anticipated attrition of 9% to give a final sample size of 600.”*

**7b) When applicable, explanation of any interim analyses and stopping guidelines**

**Does your paper address CONSORT subitem 7b?** * Yes, *“The study was terminated before its conclusion, based on guidance by an independent data safety monitoring board (DSMB), after it was found that there was no difference between study arms at 6 months. The study was closed when all participants had completed at least 12 months (follow-up period range: 12-24 months).”*

**8a) Method used to generate the random allocation sequence**

**Does your paper address CONSORT subitem 8a?** * *Yes, “Eligible patients were randomized to either the control (standard of care) or intervention arm (1:1 ratio) in this open-label study. Randomization blocks (sizes of 4) were generated by an independent statistician and kept under lock and key at the two sites. The study medical team assigned randomized participants to their final allocated study arms. We interviewed participants in the intervention arm at each visit if they had trouble using the intervention. A detailed trial design can be found in the study protocol (Multimedia Appendix 5).”*

**8b) Type of randomisation; details of any restriction (such as blocking and block size)**

**Does your paper address CONSORT subitem 8b? *** *Yes, “Eligible patients were randomized to either the control (standard of care) or intervention arm (1:1 ratio) in this open-label study. Randomization blocks (sizes of 4) were generated by an independent statistician and kept under lock and key at the two sites. The study medical team assigned randomized participants to their final allocated study arms. We interviewed participants in the intervention arm at each visit if they had trouble using the intervention. A detailed trial design can be found in the study protocol (Multimedia Appendix 5).”*

**9) Mechanism used to implement the random allocation sequence (such as sequentially numbered containers), describing any steps taken to conceal the sequence until interventions were assigned**

**Does your paper address CONSORT subitem 9? *** *Yes, “Eligible patients were randomized to either the control (standard of care) or intervention arm (1:1 ratio) in this open-label study. Randomization blocks (sizes of 4) were generated by an independent statistician and kept under lock and key at the two sites. The study medical team assigned randomized participants to their final allocated study arms. We interviewed participants in the intervention arm at each visit if they had trouble using the intervention. A detailed trial design can be found in the study protocol (Multimedia Appendix 5).”*

**10) Who generated the random allocation sequence, who enrolled participants, and who assigned participants to interventions**

**Does your paper address CONSORT subitem 10? *** Yes, ***“****The study medical team assigned randomized participants to their final allocated study arms.****”***

**11a) If done, who was blinded after assignment to interventions (for example, participants, care providers, those assessing outcomes) and how.**

**11a-i) Specify who was blinded, and who wasn’t**

**Does your paper address subitem 11a-i? *** *Yes,* *"This was an open label randomized control trial (RCT) to evaluate the impact of a patient support tool, Call for LifeTM (CFL) on QoL and viral suppression in PLHIV in Uganda. "*

**11a-ii) Discuss e.g., whether participants knew which intervention was the “intervention of interest” and which one was the “comparator”**

**Does your paper address subitem 11a-ii?** *Yes,* *"This was an open label randomized control trial (RCT) to evaluate the impact of a patient support tool, Call for LifeTM (CFL) on QoL and viral suppression in PLHIV in Uganda. "*

**11b) If relevant, description of the similarity of interventions**

**Does your paper address CONSORT subitem 11b? *** *Yes, “Participants in the intervention arm received the usual standard of care plus daily adherence IVR voice reminders (or SMS text messaging), delivered just before the usual pill-taking time. Intervention participants also received pre-appointment reminders and weekly voice calls offering educational health tips. In addition, intervention participants had an option to call a toll-free line and report symptoms or drug side effects.”*

**12a) Statistical methods used to compare groups for primary and secondary outcomes**

Does your paper address CONSORT subitem 12a? * *Yes, All analyses were conducted using Stata software, version 14 (StataCorp). We compared the changes in the QoL, and HIV RNA outcomes using Pearson chi-square or paired t-test and determined the difference in differences (DID) in the endpoints between intervention and control arms. Analysis of covariance was used to test the interaction effects of categorical variables on the QoL, controlling for the effects of other selected continuous variables, including baseline HIV RNA, CD4, and duration of care. Additionally, we compared the outcomes within the intervention arm according to the intensity of use of the system (proportion of users receiving reminders): low users (<25% calls answered), fairly low users (26%-50% of calls answered), moderate users (51%-75% answered) and high users (>75% calls answered).”*

**12a-i) Imputation techniques to deal with attrition / missing values**

Imputation techniques to deal with attrition / missing values: Not all participants will use the intervention/comparator as intended and attrition is typically high in ehealth trials. Specify how participants who did not use the application or dropped out from the trial were treated in the statistical analysis (a complete case analysis is strongly discouraged, and simple imputation techniques such as LOCF may also be problematic [4]).

**Does your paper address subitem 12a-i? *** Not applicable as no imputation methods used.

**12b) Methods for additional analyses, such as subgroup analyses and adjusted analyses**

**Does your paper address CONSORT subitem 12b? *** *Yes, “All analyses were conducted using Stata software, version 14 (StataCorp). We compared the changes in the QoL, and HIV RNA outcomes using Pearson chi-square or paired t-test and determined the difference in differences (DID) in the endpoints between intervention and control arms. Analysis of covariance was used to test the interaction effects of categorical variables on the QoL, controlling for the effects of other selected continuous variables, including baseline HIV RNA, CD4, and duration of care. Additionally, we compared the outcomes within the intervention arm according to the intensity of use of the system (proportion of users receiving reminders): low users (<25% calls answered), fairly low users (26%-50% of calls answered), moderate users (51%-75% answered) and high users (>75% calls answered).”*

**X26) REB/IRB Approval and Ethical Considerations [recommended as subheading under "Methods"] (not a CONSORT item)**

**X26-i) Comment on ethics committee approval**

Does your paper address subitem X26-i? *Yes, " The study was approved by the Makerere University School of Medicine Research Ethics Committee (REC# 2015-083) and Uganda National Council of Science and Technology and was registered with ClinicalTrials.gov (NCT02953080) [25]. All study participants provided informed consent before participation. An independent DSMB supervised the study implementation. An interim analysis was planned at 12 months a priori, and the study was to be terminated if there was no difference in the primary outcomes between study arms at 6 months”.*

**x26-ii) Outline informed consent procedures.**

Outline informed consent procedures e.g., if consent was obtained offline or online (how? Checkbox, etc.?), and what information was provided (see 4a-ii). See [6] for some items to be included in informed consent documents.

**Does your paper address subitem X26-ii?** Yes," All study participants provided informed consent for study participation”.

**X26-iii) Safety and security procedures**

Safety and security procedures, incl. privacy considerations, and any steps taken to reduce the likelihood or detection of harm (e.g., education and training, availability of a hotline)

**Does your paper address subitem X26-iii?** *Yes, " All study data were double-encrypted by CFL. All clinic data of people living with HIV remained on the local IDI servers as per Uganda data protection guidelines. Communication between the CFL browser and the server was encrypted using 128-bit Secure Sockets Layer. CFL system servers were hosted by Amazon Web Services (AWS) and secured by Amazon virtual private cloud and AWS web firewalls. At the same time, data were protected from virus threats using Bitdefender antivirus technology. "*

**RESULTS**

**13a) For each group, the numbers of participants who were randomly assigned, received intended treatment, and were analysed for the primary outcome**

NPT: The number of care providers or centers performing the intervention in each group and the number of patients treated by each care provider in each center

**Does your paper address CONSORT subitem 13a? *** Yes, This is provided in the CONSORT diagram (Figure 2).

**13b) For each group, losses and exclusions after randomisation, together with reasons**

Does your paper address CONSORT subitem 13b? (NOTE: Preferably, this is shown in a CONSORT flow diagram) *: Yes, This is provided in the CONSORT diagram (Figure 2).

**13b-i) Attrition diagram**

Strongly recommended: An attrition diagram (e.g., proportion of participants still logging in or using the intervention/comparator in each group plotted over time, similar to a survival curve) or other figures or tables demonstrating usage/dose/engagement.

**Does your paper address subitem 13b-i?** Yes, This is provided in the CONSORT diagram (Figure 2).

**14a) Dates defining the periods of recruitment and follow-up**

**Does your paper address CONSORT subitem 14a? *** *Yes, " From August 2016 to August 2017, 1079 participants were screened concurrently at the two study clinics, 715 participants were eligible, and 600 participants were enrolled (Figure 2). The most common reasons for nonenrollment included postponing enrollment to a date beyond the enrollment period—such participants were not enrolled as they returned after the sample size had been accrued (47)—and failing on second-line ART (22). The other reasons for exclusion are shown in Figure 2.* "

**14a-i) Indicate if critical “secular events” fell into the study period**

**Does your paper address subitem 14a-i?** Yes, **“** *The study was halted between January 20 and February 2, 2018, due to a software failure. There were no confidentiality breaches during this software failure, and a protocol deviation was reported to the institutional review board. Upon fixing the technical failure, participants were interviewed on whether they wanted to re-enroll. Only 1 participant out of 299 patients on the intervention arm declined (due to reasons other than software) to rejoin the study after this time. During the system failure period, all participants received the standard of care."*

**14b) Why the trial ended or was stopped (early)**

**Does your paper address CONSORT subitem 14b? *** *Yes, "At study exit (range 12 months to 24 months, due to stopping study on DSMB guidance after study demonstrated futility to primary outcome at six months)."*

**15) A table showing baseline demographic and clinical characteristics for each group**

**Does your paper address CONSORT subitem 15? *** Yes, Table 1

**15-i) Report demographics associated with digital divide issues**

**Does your paper address subitem 15-i? *** Yes, Table 1-Shows demographics by gender, age and Education.

**16) For each group, number of participants (denominator) included in each analysis and whether the analysis was by original assigned groups**

**16-i) Report multiple “denominators” and provide definitions**

Report multiple “denominators” and provide definitions: Report N’s (and effect sizes) “across a range of study participation [and use] thresholds” [1], e.g., N exposed, N consented, N used more than x times, N used more than y weeks, N participants “used” the intervention/comparator at specific pre-defined time points of interest (in absolute and relative numbers per group). Always clearly define “use” of the intervention.

**Does your paper address subitem 16-i? *** Yes please. Read the paper, all statistics have numerators and denominators.

**16-ii) Primary analysis should be intent-to-treat**

**Does your paper address subitem 16-ii?** Yes, this paper used difference in difference analysis (Table 2. Quality of life scores at baseline and 12 months.)

**17a) For each primary and secondary outcome, results for each group, and the estimated effect size and its precision (such as 95% confidence interval)**

**Does your paper address CONSORT subitem 17a?** * Yes, please see manuscript results.

**17a-i) Presentation of process outcomes such as metrics of use and intensity of use**

In addition to primary/secondary (clinical) outcomes, the presentation of process outcomes such as metrics of use and intensity of use (dose, exposure) and their operational definitions is critical. This does not only refer to metrics of attrition (13-b) (often a binary variable), but also to more continuous exposure metrics such as “average session length”. These must be accompanied by a technical description how a metric like a “session” is defined (e.g., timeout after idle time) [1] (report under item 6a).

**Does your paper address subitem 17a-i?** Yes. See Figure 3

**17b) For binary outcomes, presentation of both absolute and relative effect sizes is recommended.**

**Does your paper address CONSORT subitem 17b? *** Not applicable

**18) Results of any other analyses performed, including subgroup analyses and adjusted analyses, distinguishing pre-specified from exploratory**

**Does your paper address CONSORT subitem 18? *** Yes, Table 4. Change in quality of life by utilization of the intervention."

**18-i) Subgroup analysis of comparing only users**

**Does your paper address subitem 18-i?** Yes, lease see table 4.

**19) All important harms or unintended effects in each group**

**Does your paper address CONSORT subitem 19? *** Not applicable-There were no anticipated or actual harms related to this intervention

**19-i) Include privacy breaches, technical problems**

Include privacy breaches, technical problems. This does not only include physical “harm” to participants, but also incidents such as perceived or real privacy breaches [1], technical problems, and other unexpected/unintended incidents. “Unintended effects” also includes unintended positive effects [2].

**Does your paper address subitem 19-i?** *" The study was halted between January 20 and February 2, 2018, due to a software failure. There were no confidentiality breaches during this software failure, and a protocol deviation was reported to the institutional review board. Upon fixing the technical failure, participants were interviewed on whether they wanted to re-enroll. Only 1 participant out of 299 patients on the intervention arm declined (due to reasons other than software) to rejoin the study after this time. During the system failure period, all participants received the standard of care”.*

**19-ii) Include qualitative feedback from participants or observations from staff/researchers**

Include qualitative feedback from participants or observations from staff/researchers, if available, on strengths and shortcomings of the application, especially if they point to unintended/unexpected effects or uses. This includes (if available) reasons for why people did or did not use the application as intended by the developers.

**Does your paper address subitem 19-ii?** This is not the focus of this current paper. Qualitative results will be published in future papers.

**DISCUSSION**

**22) Interpretation consistent with results, balancing benefits and harms, and considering other relevant evidence**

**22-i) Restate study questions and summarize the answers suggested by the data, starting with primary outcomes and process outcomes (use)**

Restate study questions and summarize the answers suggested by the data, starting with primary outcomes and process outcomes (use).

**Does your paper address subitem 22-i? *** *YES, To our knowledge, this is the largest mHealth intervention trial to evaluate the impact of IVR technology and the only one to offer a choice of IVR and SMS text messaging in Africa. Mobile health technologies that utilize voice calls are ideal for patient support in most LMICs due to high illiteracy rates [26]. Our study did not find any statistical difference in the change in QoL at 12 months in participants enrolled in the intervention and control arms. Similarly, there was no difference in viral suppression rates in the two arms. However, we found an association between improved QoL (overall and MHS) as well as viral suppression and adherence to clinic appointments in participants who had moderate or high use of the tool.*

**22-ii) Highlight unanswered new questions, suggest future research**

**Highlight unanswered new questions, suggest future research.**

**Does your paper address subitem 22-ii?** *Yes “The software did not find a significant difference in appointment keeping, but most of the patients were highly experienced. Therefore, the intervention should be evaluated in people living with HIV who are newly engaging in care."*

**20) Trial limitations, addressing sources of potential bias, imprecision, and, if relevant, multiplicity of analyses**

**20-i) Typical limitations in ehealth trials**

**Does your paper address subitem 20-i? *** *Yes, "There were limitations in this study. Whilst attempting to increase generalizability of our study findings, we enrolled heterogeneous populations and included patients who were highly experienced on ART as well as patients newly diagnosed and initiated on ART. Due to the successful ART scale-up, PLHIV are increasingly healthier at diagnosis [23,24] and may have higher QoL scores, and those established on ART also have higher QOL scores.*

**21) Generalisability (external validity, applicability) of the trial findings**

**NPT: External validity of the trial findings according to the intervention, comparators, patients, and care providers or centers involved in the trial.**

**21-i) Generalizability to other populations**

Generalizability to other populations: In particular, discuss generalizability to a general Internet population, outside of a RCT setting, and general patient population, including applicability of the study results for other organizations.

**Does your paper address subitem 21-i?** Yes, "In the prevailing circumstances when most developing countries are under lockdown and most PLHIV do not have physical support, IVR could provide alternative support. The impact in such situation needs to be evaluated. "

**21-ii) Discuss if there were elements in the RCT that would be different in a routine application setting.**

Discuss if there were elements in the RCT that would be different in a routine application setting (e.g., prompts/reminders, more human involvement, training sessions or other co-interventions) and what impact the omission of these elements could have on use, adoption, or outcomes if the intervention is applied outside of a RCT setting.

**Does your paper address subitem 21-ii?** Not applicable

**OTHER INFORMATION**

**23) Registration number and name of trial registry**

**Does your paper address CONSORT subitem 23?** * Yes, we provide the the URL for clinical trial registry "ClinicalTrials.gov NCT02953080; https://clinicaltrials.gov/ct2/show/NCT02953080, "

**24) Where the full trial protocol can be accessed, if available**

**Does your paper address CONSORT subitem 24? ***Annexed to this submission.

**25) Sources of funding and other support (such as supply of drugs), role of funders**

**Does your paper address CONSORT subitem 25? ***

Yes. "Janssen, the Pharmaceutical Companies of Johnson & Johnson provided funding for this study and reviewed the study design. However, the funders did not participate in study conduct, statistical analysis, or manuscript writing."

**X27) Conflicts of Interest (not a CONSORT item)**

**X27-i) State the relation of the study team towards the system being evaluated.**

In addition to the usual declaration of interests (financial or otherwise), also state the relation of the study team towards the system being evaluated, i.e., state if the authors/evaluators are distinct from or identical with the developers/sponsors of the intervention.

**Does your paper address subitem X27-i? Yes,** "Janssen, the Pharmaceutical Companies of Johnson & Johnson provided funding for this study and reviewed the study design. However, the funders did not participate in study conduct, statistical analysis, or manuscript writing."

**Multimedia Appendix 6b:** Compliance to the mHealth evidence reporting and assessment (mERA) guidelines

| **Criteria** | **Item no** | **Notes** | **The page where the item is reported** |
| --- | --- | --- | --- |
| Infrastructure (population level) | 1 | Clearly presents the availability of infrastructure to support technology operations in the study location. This refers to physical infrastructure such as electricity, access to power, connectivity etc. in the local context. Reporting X% network coverage rate in the country is insufficient if the study is not being conducted at the country level | page 9 |
| Technology | 2 | Describes and justifies technology architecture. This includes a description of software and hardware and details of any modifications made to publicly available software | page 9 |
| platform |  |  |  |
| Interoperability/Health information systems (HIS) context | 3 | Describes how mHealth intervention can integrate into existing health information systems. Refers to whether the potential of technical and structural integration into existing HIS or program has been described irrespective of whether such integration has been achieved by the existing system | page 8, 11 |
| Intervention delivery | 4 | The delivery of the mHealth intervention is clearly described. This should include frequency of mobile communication, mode of delivery of the intervention (that is, SMS, face to face, interactive voice response), timing and duration over which delivery occurred | page 9 |
| Intervention content | 5 | Details of the content of the intervention are described. Source and any modifications of the intervention content is described | page 9 |
| Usability/content testing | 6 | Describe formative research and/or content and/or usability testing with the target group(s) clearly identified, as appropriate | 8 |
| User feedback | 7 | Describes user feedback about the intervention or user satisfaction with the intervention. User feedback could include user opinions about content or user interface, their perceptions about usability, access, connectivity, etc. | 14 (qualitative research is not presented in this paper) |
| Access of individual participants | 8 | Mentions barriers or facilitators to the adoption of the intervention among study participants. Relates to individual-level structural, economic, and social barriers or facilitators to access such as affordability, and other factors that may limit a user’s ability to adopt the intervention | 16 |
| Cost assessment | 9 | Presents basic costs assessment of the mHealth intervention from varying perspectives. This criterion broadly refers to the reporting of some cost considerations for the mHealth intervention in lieu of a full economic analysis. If a formal economic evaluation has been undertaken, it should be mentioned with appropriate references. Separate reporting criterion are available to guide economic reporting | 17 |
| Adoption inputs/ program entry | 10 | Describes how people are informed about the program including training, if relevant. Includes description of promotional activities and/or training required to implement the mHealth solution among the user population of interest | 10 |
| Limitations for delivery at scale | 11 | Clearly presents mHealth solution limitations for delivery at scale | 17 |
| Contextual adaptability | 12 | Describes the adaptation, or not, of the solution to a different language, different population, or context. Any tailoring or modification of the intervention that resulted from pilot testing/usability assessment is described | 8 |
| Replicability | 13 | Detailed intervention to support replicability. Clearly presents the source code/screenshots/ flowcharts of the algorithms or examples of messages to support replicability of the mHealth solution in another setting | Multimedia Appendix figure 1 |
| Data security | 14 | Describes the data security procedures/ confidentiality protocols | 11 |
| Compliance with national guidelines or regulatory statutes | 15 | The mechanism used to assure that content or other guidance/information provided by the intervention is in alignment with existing national/regulatory guidelines and as described | 11 |
| Fidelity of the intervention | 16 | Was the intervention delivered as planned? Describe the strategies employed to assess the fidelity of the intervention. This may include assessment of participant engagement, use of backend data to track message delivery and other technological challenges in the delivery of the intervention | 13 |
